# Supplementary material for: Modelling factors in primary care quality improvement: a cross-sectional study of premature CHD mortality
Source: BMJ Open. 2013 Oct 22;3(10):e003391. doi: 10.1136/bmjopen-2013-003391 (PMC3808822; doi:10.1136/bmjopen-2013-003391)
Supplement: Author's manuscript [file bmjopen-2013-003391.draft_revisions.pdf]

Modelling factors in primary care affecting premature coronary heart disease mortality: a cross sectional study.

Journal:	*BMJ Open*
Manuscript ID:	bmjopen-2013-003391
Article Type:	Research
Date Submitted by the Author:	11-Jun-2013
Complete List of Authors:	Honeyford, Kate; University of Leicester, Health Sciences<br>Baker, Richard; University of Leicester, Health Sciences<br>Bankart, M. John; Keele University, Institute of Primary Care<br>Jones, David; University Of Leicester, Health Sciences
**Primary Subject Heading**:	Public health
Secondary Subject Heading:	Health services research, Epidemiology, Cardiovascular medicine
Keywords:	PRIMARY CARE, STATISTICS & RESEARCH METHODS, Cardiac Epidemiology < CARDIOLOGY, Coronary heart disease < CARDIOLOGY

SCHOLARONE™
Manuscripts

For peer review only

TITLE PAGE

Title: Modelling factors in primary care affecting premature coronary heart disease mortality: a cross sectional study.

Corresponding author:

Kate Honeyford MSc

Adrian Building,

University Road,

Leicester

LE1 7RH

ceh28@le.ac.uk

Tel: 0116 229 7254/7255

Fax: 0116 229 7250

Co-authors:

Professor Richard Baker, Dept of Health Sciences, University of Leicester, Leicester, UK.

Dr M. John G. Bankart, Insitute of Primary Care and Health Sciences, Keele University, Keele, UK.

Professor David Jones, Dept Health Sciences, University of Leicester, Leicester, UK.

Key words:

Primary health care

Regression analysis

Mortality, premature

Coronary disease

Cross-sectional studies

Word count: 3995 (including section headings)

For peer review only

For peer review only - http://bmjopen.bmj.com/site/about/guidelines.xhtml

ABSTRACT

Objectives

i) To identify features of primary care associated with reduced premature coronary heart disease (CHD) mortality, and

ii) to determine the impact of different modelling approaches.

Design

Cross sectional study of mortality rates in 229 general practices.

Setting

General practices from three East Midlands primary care trusts.

Participants

Patients registered to practices above between April 2006 and March 2009.

Main outcome measures

Numbers of CHD deaths in those aged under 75 and at all ages in each practice.

Results

Both population characteristics and markers of quality of primary care were associated with variations in premature CHD mortality. Increasing levels of deprivation, and the percentage of the practice population on the practice diabetes register, white, over 65 and male were all associated with increasing levels of premature CHD mortality. Control of serum cholesterol levels in those with CHD and the percentage of patients recalling access to their preferred GP were both associated with decreased levels of premature CHD mortality. Similar results were found for all age mortality. A combined measure of quality of primary care with regards to CHD comprising 12 QOF indicators was associated with decreases in both all age and premature CHD mortality. The selected models suggest that practices in less deprived areas may have up to 20% lower premature CHD mortality than those with median deprivation and that improvement in the CHD quality of care measure from 83% (lower quartile) to 86% (median) could reduce premature CHD mortality by 3.6%. Different modelling approaches yielded qualitatively similar results.

Conclusions

High quality primary care, including aspects of access to and continuity of care, detection and management, appears to be associated with reducing CHD mortality. The impact on premature CHD mortality is greater than on all-age CHD mortality. Determining the most useful measures of quality of primary care needs further consideration.

For peer review only - http://bmjopen.bmj.com/site/about/guidelines.xhtml

For peer review only

Article Focus:

- CHD is a major cause of premature deaths in the UK; primary care can play a role in modifying risk factors in populations.
- This study identifies features of primary care associated with lower levels of premature coronary heart disease.
- A variety of methods have been used to model associations; this paper explores the impact of different modelling approaches.

Key Messages:

- High quality primary care, as measured through QOF clinical indicators and relational continuity, are associated with lower levels of premature CHD mortality.
- To inform future monitoring and development of primary care, more reliable data relating to smoking and other lifestyle factors is necessary.
- Results and their interpretation show some sensitivity to modelling approaches.

Strengths and weaknesses

- The associations between several modifiable risk factors and premature CHD mortality are considered, and compared to those for all age CHD mortality.
- The study examines the impact of model choice and the measure of primary care quality on results and interpretation.
- The lack of reliable data relating to lifestyle factors such as smoking and obesity rates mean that important covariates are not included in the model.

For peer review only

For peer review only - http://bmjopen.bmj.com/site/about/guidelines.xhtml

INTRODUCTION

In England, in common with health systems in many other countries, health outcomes are increasingly being used to plan and manage health services.[1] A framework for health outcomes has been introduced to provide an overview of how the national health service (NHS) is performing and as a catalyst for driving quality.[2] One of the framework's five domains is the prevention of premature death, one of the twelve indicators in this domain being death from cardiovascular disease under the age of 75 years.

Whilst mortality caused by coronary heart disease (CHD) has been falling steadily since the 1980s[3] it is still the leading cause of years of life lost in the UK.[4] A recent report based on the Global Burden Disease Study shows that CHD mortality rates in the UK are significantly above the mean of the 15 original members of the European Union and four other comparator countries. Although population characteristics including lifestyle (eg obesity, smoking) and hypertension are known to be associated with CHD mortality, primary care may have a role in reducing CHD mortality. For example, in the US, greater supply of primary care physicians is associated with lower heart disease mortality,[5] although there is little evidence of a similar association in the UK.[6] Since general practices serve lists of registered patients, there is potential to relate mortality in these practice populations to both population characteristics and the performance of practices, and thereby assist practices in planning and monitoring their activities to reduce population mortality.[7] A conceptual model to describe how the delivery of primary care can modify the impact of population based characteristics on health outcomes has been proposed.[8] The model includes interventions which target both the morbid and healthy populations via early detection, prevention and appropriate management of people with established disease and highlights the importance of access and continuity of care. However, the size of practice populations and the small numbers of deaths present methodological difficulties.[9] In seeking to understand how primary care performance affects mortality in practice populations, consideration must be given to **(1)** the approach to measurement of the performance of primary care; and **(2)** selection of the underlying statistical model.

In recent years, information about the performance of general practices has become available, in addition to other practice characteristics such as numbers of registered patients and numbers of general practitioners. The Quality Outcomes Framework (QOF) has provided data in four domains (clinical; organisational; patient experience and additional). Within the clinical domain are several chronic conditions (including coronary heart disease), each made up of individual indicators. For each indicator points are awarded to practices based on the percentage of patients for whom the target has been achieved (known as the underlying achievement). Both the score and the underlying achievement for each indicator are publicly available. Additionally, QOF contains 'prevalence registers' that show the percentage of the practice population identified by the practice as having a particular condition or disease.

In investigating aspects of practice performance associated with various outcomes, Kiran et al used combinations of individual indicators' underlying achievement to give a devised 'CHD quality achievement score'.[10]

For peer review only - http://bmjopen.bmj.com/site/about/guidelines.xhtml

QOF prevalence registers have also been used as a measure of quality of primary care; for example, in a study at the level of primary care trusts, greater numbers of people on practice hypertension registers have been shown to be associated with lower CHD mortality, indicating that improved detection of hypertension has a positive impact on outcome.[8 11]

The different ways in which QOF data have been used may partly explain the differences found in different studies. For example, Kiran et al [10] found that the overall CHD quality achievement score was significantly negatively associated with CHD mortality. However, other studies have shown no association between *individual* indicators and CHD mortality [8] and CHD admissions [12] or overall scores and emergency and elective admissions. [13 14]

Statistical Method and Model Selection

Different statistical models may be used. A common method is multiple linear regression with age-standardised mortality rates as the dependent variable. However, since in research of this type age-adjusted variables are rarely available multiple linear regression of standardised mortality rates can lead to biased risk estimates.[15–17]

Poisson models have the advantage of considering deaths as count data; age and sex can then be included in the model as explanatory variables, thus overcoming the lack of age standardised explanatory variables. As the counts are often over-dispersed, a negative binomial model may be more appropriate than a Poisson model.[18] Models based on count data, for example the numbers of deaths or hospital admissions, can also be found in the literature.[8 13 19–21]

Premature Mortality

Premature mortality may be defined in various ways, although the Outcomes Framework adopts a definition of deaths under age 75, in accordance with the preference of the Office of National Statistics.[22] Few studies have considered the associations between quality of primary health care, as measured by QOF, and *premature* mortality. Allender et al[23] demonstrated a stronger association between socio-economic deprivation and premature CHD mortality, compared to all-age CHD mortality. In a study of CHD hospitalisation and primary care[12] patients were divided into 45-74 and 75 and over; socio-economic status was found to be more important in the younger age group, but no difference was found in the association of quality of care and health outcomes in the two different age groups.

In this study, we investigate the choice of performance indicators and statistical models to explain all-age and premature practice population mortality from coronary heart disease. The overall aim is to explore approaches with potential to help practices plan strategies to reduce population mortality.

For peer review only - http://bmjopen.bmj.com/site/about/guidelines.xhtml

METHODS

This is an observational study of CHD mortality between April 2006 and March 2009 in 229 practices in the East Midlands. All 230 practices that were open for the three years of the study were eligible for inclusion. One practice offering a service to a restricted patient group was excluded from the study. The total population covered by the study is just under 1.7 million people. In this study premature CHD mortality will be the main outcome measure; this will be compared with all-age CHD mortality. Counts of deaths will be modelled using negative binomial regression. Results will be compared to those from weighted linear regression of indirectly standardised mortality ratios to determine the impact of the model adopted on the overall findings and conclusions of the analysis.

CHD mortality counts for each practice were constructed from the Primary Care Mortality Database for the period, supplied by the relevant PCTs. Each record included date of birth, date of death, underlying cause of death and the general practice code for the patient's practice. Each death was linked to a general practice using these codes. Deaths with underlying cause identified as CHD (ICD-10 codes: I20-I25) were included in the study. In addition, indirectly standardised mortality rates (SMR) were calculated for each practice, based on England and Wales' standard rates and midyear estimates of practice populations provided by PCTs in line with NCHOD's methodology.[24] Since numbers of deaths in each practice per year were low, both counts and SMRs were aggregated over the three years.

QOF clinical data are publicly available for all practices in the sample for 2006/07, 2007/08 and 2008/09.[25] The 2006/7 indicators were used since the primary care described by indicators for that year will have had an impact on the largest proportion of patients included in the analysis. Levene et al[8] classify primary healthcare into prevention, early detection and appropriate management. QOF indicators can be classified following this approach:

Prevention: Prevention of smoking and obesity. Reducing the number of people who are smoking or are obese would be useful indicators of prevention; however neither of these is available for practice populations. The QOF indicator which details the percentage of patients having a range of conditions who have been offered smoking cessation advice is being used as a measure of prevention of smoking (SM02).

Early detection: Detection of hypertension. Levene et al [8 11] argue that given only a little more than half of the people found to have hypertension in population surveys in England are included on QOF hypertension registers these can be used as a useful measure of hypertension detection.

Appropriate disease management: Indicators identified by Levene et al have been selected for this analysis:- **(a)** the control of serum cholesterol in patients on practice CHD registers (CHD08) and **(b)** the percentage of the practice CHD register who are being treated with aspirin (or equivalent) (CHD09). See Table 1 for more detailed descriptions of QOF indicators.

The CHD quality achievement score devised by Kiran et al[10] is included in a comparative analysis. The measure is the mean underlying achievement of 12 indicators (see details in Table 1).

Access and sustained relationships: one question from the General Practice Patient Survey[26] relating to patients' recall of being able to consult a particular GP has been selected as an indicator of the ability to access care and whether patients prefer to have access to a sustained relationship in line with the work of Levene et al.[8] In addition, the number of GPs per 1000 patients is being included as a measure of GP supply.

For peer review only - http://bmjopen.bmj.com/site/about/guidelines.xhtml

Characteristics of the **practice population** have been selected based on previous research, the use of count data and availability. The percentage of the practice population who are male and are aged over 65 are included because they are known to be associated with higher rates of CHD mortality. List size is included as a measure of exposure in the negative binomial model, to take into account that practices vary in size and therefore the number at risk varies from practice to practice. List size is included in multiple regression for consistency. Diabetes prevalence, ethnicity and deprivation are included as previous research has shown these to be important in explaining variation in CHD mortality ([8 11], [10] and [8 10] respectively). Neither obesity nor smoking rates are included as the measures available do not accurately describe the rates within practice populations. The sources of information used for each of these are summarised in Table 2.

Eight practices have missing data for the number of GPs (fte); three practices have missing data from the GP Patient survey. The main analysis was repeated using a range of values; choice of value was not important. Either the median value or values from following years have been used.

Statistical analysis

Counts of deaths in each practice are modelled here using negative binomial regression in preference to Poisson regression since the data are over-dispersed (over-dispersion parameter $\alpha=0.023$, $p<0.05$). To explore how sensitive the results and interpretation are to the modelling approach used, ordinary least squares (OLS) linear regression with the standardised mortality ratio (SMR) as the dependent variable was also carried out, using the inverse of the variance of SMR as a weighting to account for uncertainty in the standardised mortality rates, in line with the work of Kiran et al.[10]

For peer review only

For peer review only - http://bmjopen.bmj.com/site/about/guidelines.xhtml

RESULTS

Table 3 shows summary data of counts of death, mortality rates, and characteristics of both practice populations and primary care for the 229 practices, serving approximately 1.7 million patients in 2006/07. Table 4 shows the estimates of incident rate ratios (IRRs) in the presented negative binomial model, including 95% confidence intervals and *p* values for all explanatory variables; the impact of a one unit increase in each explanatory variable is also included to aid the interpretation of IRRs. As in many models in this field reporting pseudo R^2, adjusted pseudo R^2 is low, although the predicted and observed counts are relatively close (Lin's concordance coefficient : 0.86). There is no pattern in the residuals and removal of identified outliers did not materially affect interpretation of the model.

Table 5 shows the potential impact that changes in population or service characteristics could have on premature CHD mortality, given the current model. For each explanatory variable the change from either the upper or lower quartile to the median is used to show how decreases in the 'risk' in the practice population or increases in the quality of primary care could impact on mortality, if the model is adequate and relationships causal.

Sensitivity analysis explored the impact of various smoking and obesity indicators. Inclusion of QOF registers or modelled estimates for local area obesity prevalence do not materially change the coefficients; there is no evidence that the model is improved by so doing. Similar results are found when both modelled estimates of smoking rates and QOF indicators relating to smoking are included. None of these indicators has been included in the final model.

Population Characteristics

The main predictors of variation in premature CHD mortality of the population characteristics included in the model were deprivation, age (percentage aged 65 and over), the percentage on the diabetes register and the percentage male. Table 5 suggests that practices in less deprived areas may have up to 20% lower premature CHD mortality counts than those with deprivation at the median.

Service Characteristics

Increases in levels of service in terms of the percentage of the CHD register whose serum cholesterol is controlled (CHD08), and the percentage of patients who recalled being able to see their preferred GP, were associated with lower levels of premature CHD mortality, as was (less clearly) increased hypertension detection (as measured through QOF hypertension prevalence registers).

Similarly, there is a suggestion that increases in GPs per 1000 patients and the percentage of the CHD register being treated with aspirin (or an alternative) are both associated with higher premature mortality. Table 5 allows interpretation of these key variables in the current model, and suggests that increasing the percentage of patients who recall being able to see their preferred GP from a lower performing practice (typically 80%) to a median performing practice (88%) may reduce premature CHD mortality counts by 4.8%. Similarly, increasing the percentage of patients on the CHD register for whom their serum cholesterol is below 5mmol/l from a low performing practice (78%) to a median performing practice (82%) may reduce premature CHD mortality counts by 3.6%.

For peer review only - http://bmjopen.bmj.com/site/about/guidelines.xhtml

Effect of choice of measure of quality of primary care

Similar results are found when the Kiran CHD quality achievement score is used in the model instead of the two separate CHD quality indicators. In the subsequent model an improvement from 83% (lower quartile) mean achievement in the Kiran overall CHD achievement score to 86% (median) reduces CHD mortality counts by 3.6% (see Appendix 1 for more information).

Effect of model selection

When a weighted multiple linear regression model for standardised mortality ratio was used, the directions of associations were the same as those described above. However, the confidence intervals for the β-coefficients for both prevalence of diabetes and the CHD quality achievement measures now include zero, and hence interpretation of the results might be less clear cut (see Appendix 2 for more information).

Comparison with all age mortality

The results for all age CHD mortality are broadly similar to those for premature CHD mortality. Increases in white ethnicity, deprivation and diabetes prevalence and an increase in GPs per 1000 patients are associated with increases in mortality, whereas improvements in the remaining service characteristics correspond to decreases in mortality counts. However, the confidence intervals for the IRRs for all service characteristics include one, impacting on the interpretation of the importance of these variables.

DISCUSSION

Statement of principal findings

Both population characteristics and the quality of primary care were found to be associated with variations in premature CHD mortality. Increasing levels of deprivation, the percentage of the practice population who were on the practice diabetes register, who were white, over 65 and who were male were all associated with increasing levels of premature CHD mortality. Control of serum cholesterol levels in those with CHD and the percentage of patients who could recall being able to see their preferred GP were both associated with decreased levels of premature CHD mortality. Similar results were found when all age mortality was considered. The combined measure of the quality of primary care was associated with a decrease in both all age and premature CHD mortality. However, it is difficult to determine which individual indicators within this measure are key to reducing CHD mortality. The evidence that hypertension detection is associated with decreased CHD mortality is less clear than has been found by Levene et al at PCT level.[8] Different modelling approaches yielded qualitatively similar results; however, detailed interpretation of the results would be model dependent, particularly if statistical significance were rigidly applied as a criterion of importance.

Strengths and weaknesses of this study

A key strength of this study is that it considers the association of features of primary care with *premature* CHD mortality, and whilst the overall relationships are similar to those found when all age

For peer review only - http://bmjopen.bmj.com/site/about/guidelines.xhtml

mortality is considered, some associations are stronger, for example, that with patients' recall of being able to see their preferred GP.

Most explanatory variables in this study describe the adult practice population and not those under 75. To develop our understanding of the different contributions made by different variables in explaining premature mortality it would be helpful to have reliable data describing different age groups within practice populations.

This study allows useful consideration of the impact of the measure of primary care quality used. Whilst the higher underlying achievement in overall CHD achievement score devised by Kiran is associated with decreased premature mortality, this doesn't allow policy makers or clinicians to determine which of the 12 indicators are most important. The examination of the individual indicators shows that increasing the percentage of patients on the CHD register whose serum cholesterol is below 5mmol/l is associated with decreasing mortality, but it is not clear whether this is the most important indicator. Unfortunately, as patient level data relating to QOF indicators are not available for this study, it is not possible to determine whether combinations of indicators within individuals are key or if there are interactions between indicators and other characteristics of individuals, for example ethnicity or gender.

Reliable information about smoking rates, alcohol consumption and obesity within practice populations is lacking, and having access to this data would be likely to improve the model fit. Whilst various estimates are available, many of these are modelled estimates for geographical areas, not practice populations, based on levels of deprivation, ethnicity and age, which are anyway included in the models in this study. The introduction of new smoking indicators in the 2012/13 QOF asks practices to record the smoking status of those aged 15 years and over and to record an offer of support and treatment to those who smoke, which may start to give a fuller picture of smoking prevalence in practice populations. Whilst a QOF smoking indicator relating to smoking cessation advice has been included in this study as a measure of prevention, the wording asks only for 'an offer of support and treatment' which may throw doubt on its validity.[27]

This study allows the impact of statistical method and model selection to be considered. Whilst the directions of associations are generally not affected by the choice of model the statistical significance of the results vary between models for several key variables. For example, neither the prevalence of diabetes nor the CHD quality achievement score would be considered to be significantly associated with premature mortality using a weighted linear regression model for SMRs.

Relation to other studies

Previous research has shown that primary care is important in improving health outcomes but that the precise aspects of primary care which are most important are not clear.[8 10 13 14 28] This study confirms that position, but explores other aspects of the problem.

An association between continuity of care and reduced mortality in older patients has been found in the US[29] and Bankart et al[14] have shown that in practices with higher mean rates of satisfaction with being able to consult a preferred GP, emergency admissions are lower. These findings give further support to the importance of continuity of care in improving health outcomes.

For peer review only - http://bmjopen.bmj.com/site/about/guidelines.xhtml

Levene et al have found that hypertension detection was associated with reduced levels of CHD mortality in two studies completed at PCT level. In their recent analysis of UK health performance based on the Global Burden of Disease Study 2010 Murray et al[4] highlight the importance of early detection and long term management of high blood pressure as a 'clear route to accelerate progress for the leading causes of avoidable cardiovascular mortality'. However, the evidence of the importance of hypertension detection in this study is not clear cut. It may be that QOF prevalence registers are not a useful measure of detection for individual practices. Low QOF prevalence rates may be due to lower levels of hypertension rather than lower detection and conversely high QOF prevalence rates may be due to higher underlying prevalence rather than improved detection. More work to identify the most useful combination of QOF indicators to summarise hypertension detection is necessary.
In line with other studies, socio-economic deprivation is the main predictor of CHD mortality. Both Bottle[12] and Allender[23] have found that socio-economic deprivation is more strongly associated with mortality in younger age groups and this study has confirmed this pattern.

Whilst some studies clearly state the reasons for their selection of statistical modelling approach and/or potential explanatory variables (for example [13 14 19 30 31]), many studies do not. Here we find some indication that qualitative interpretations of results are robust to model choice. However, more detailed interpretation of results is likely to vary between models, particularly if there is undue reliance on simplistic interpretation of statistical significance.

Meaning of the study

This study adds to the body of research demonstrating that high quality primary care is associated with improving health outcomes. Aspects of both continuity of care and disease management have been identified as having a bigger impact on reducing premature CHD mortality than all-age CHD mortality. Whilst the most important individual indicators relating to disease management have not been identified, there is clear evidence that improving achievement in QOF indicators is associated with decreasing CHD mortality. The importance of continuity of care, again shown here, strongly suggests that this is an area for general practices to prioritise. The findings also suggest that data about outcomes such as premature CHD mortality could be used by practices to monitor and, over several years, plan their care to improve population health.

The ongoing importance of socio-economic deprivation in explaining higher levels of mortality cannot be ignored. Understanding the relationship between deprivation and health outcomes more precisely remains an important area of further study.

Unanswered questions and future research

The lack of reliable practice level information on key areas such as obesity, alcohol and smoking prevalence has an important impact on primary care research and is an important health information issue needing effective attention as the NHS undergoes major changes. Whilst QOF indicators relating to smoking prevalence have been introduced, the reliability of these measures will need to be scrutinised.

For peer review only - http://bmjopen.bmj.com/site/about/guidelines.xhtml

For peer review only

To further our understanding of the relative importance of different QOF indicators it would be useful to study individual QOF indicators at patient level and how they interact with each other and with characteristics of individuals on an individual patient basis.

CONCLUSION

Improving the quality of primary care will play an important part in decreasing premature mortality, and there is evidence that high underlying achievements in QOF clinical indicators are a useful measure of quality primary care. Continuity of care, in a country with universal access to healthcare is important and should not be underestimated by policy makers and clinicians. Nonetheless lifestyle factors are also important, but our ability to study them adequately in primary care, or to evaluate the role of primary care in addressing them, is currently limited by the quality of measures of them at practice level. If primary care services, delivered by clinical commissioning groups are to be monitored and developed using the new NHS outcomes Framework, better data and careful modelling and interpretation are vital.

For peer review only

For peer review only - http://bmjopen.bmj.com/site/about/guidelines.xhtml

Tables

Table 1: QOF indicators used to compile the overall CHD quality achievement score.

Clinical domain			Summary statistics in this study (median and interquartile range)
**CHD**	CHD06	The percentage of patients with CHD in whom the last blood pressure reading (measured in the previous 15 months) is ≤150/90 mm Hg.	90 (85, 93)
	CHD08	The percentage of patients with CHD whose last measured total cholesterol level (measured in the previous 15 months) is ≤5 mmol/l.	82 (78, 87)
	CHD09	The percentage of patients with CHD with a record in the previous 15 months that aspirin, an alternative antiplatelet therapy, or an anti-coagulant is being taken (unless a contraindication or adverse effects are recorded).	95 (93, 97)
	CHD10	The percentage of patients with CHD who are currently treated with a β-blocker (unless a contraindication or adverse effects are recorded).	73 (66, 81)
	CHD11	The percentage of patients with a history of myocardial infarction (diagnosed after 1 April 2003) who are currently treated with an angiotensin-converting enzyme inhibitor or angiotensin II antagonist.	92 (88, 97)
**Stroke and TIA**	STROKE06	The percentage of patients with TIA or stroke in whom the last blood pressure reading (measured in the previous 15 months) is ≤150/90 mm Hg.	88 (82, 92)
	STROKE08	The percentage of patients with TIA or stroke whose last measured total cholesterol level (measured in the previous 15 months) is ≤5 mmol/l.	76 (70, 83)
**Hypertension**	BP05	The percentage of patients with hypertension in whom the last blood pressure reading (measured in the previous 9 months) is ≤150/90 mm Hg.	77 (73, 83)
**Diabetes mellitus**	DM12	The percentage of patients with diabetes in whom the last blood pressure reading is ≤145/85 mm Hg.	80 (74, 85)
	DM17	The percentage of patients with diabetes whose last measured total cholesterol level (measured in the previous 15 months) is ≤5 mmol/l.	83 (77, 87)
	DM20	The percentage of patients with diabetes in whom the last HbA1C is ≤ 7.5 (or equivalent test/reference range depending on laboratory) in the previous 15 months.	67 (61, 73)
**Smoking**	SMOKE02	The percentage of patients with any or any combination of the following condition: CHD, stroke or TIA, hypertension, diabetes, COPD or asthma, who smoke and whose notes contain a record that smoking cessation advice or referral to a specialist service, where available, has been offered within the previous 15 months.	94 (92, 96)
**Kiran CHD overall achievement score**		Mean of the above 12 indicators	83 (80, 86)

Table 2: Characteristics of practice populations.

Measure	Source of data
**Deprivation indices**	Based on Index of Multiple Deprivation 2007.
**% of GP list on diabetes register**	QOF prevalence register 2006/07.
**% White ethnicity**	Based on hospital episode statistics.
**% of population who are over 65**	Based on mid-year practice population estimates provided by the PCT.
**% of population who are male**	Based on mid-year practice population estimates provided by the PCT.
**List size**	Based on mid-year practice population estimates provided by the PCT.

For peer review only - http://bmjopen.bmj.com/site/about/guidelines.xhtml

Table 3 Summary statistics for mortality counts, population characteristics and service characteristics.

	Summary statistics in this study Median (IQR)
**Mortality data**	
Indirectly Standardised CHD mortality	95.9 (78.3, 119.9)
Indirectly Standardised CHD mortality – under 75	92.4 (64.0, 135.6)
Crude rate per 1000	1.30 (0.95, 1.57)
Crude rate per 1000 – under 75	0.43 (0.28, 0.58)
**Practice characteristics**	
Deprivation indices	16.2 (10.0, 27.0)
% of GP list on diabetes register	3.8 (3.3, 4.4)
% White ethnicity	89.9 (77.5, 94.1)
% of population who are over 65	14.7 (12.1, 17.0)
% of population who are male	50.2 (49.5, 51.2)
List size	6435 (3749, 10319)
**Service characteristics**	
GPs per 1000	0.55 (0.47, 0.64)
% patients with recalled perception of being able to see preferred GP	88 (80, 93)
% of GP registered list on hypertension register	12.3 (11.0, 14.7)
The percentage of patients with any or any combination of the following conditions: coronary heart disease, stroke or TIA, hypertension, diabetes, COPD or asthma who smoke whose notes contain a record that smoking cessation advice or referral to a specialist service, where available, has been offered within the previous 15 months. SM02	94 (92, 96)
The percentage of patients with CHD whose last measured total cholesterol level (measured in the previous 15 months) is ≤5 mmol/l. CHD08	82 (78, 87)
The percentage of patients with CHD with a record in the previous 15 months that aspirin, an alternative antiplatelet therapy, or an anti-coagulant is being taken (unless a contraindication or adverse effects are recorded). CHD09	95 (93, 97)
CHD overall achievement score[10]	83 (80, 86)

For peer review only

Table 4 Estimated incident rate ratios, associated 95% confidence intervals and associated *p* values for negative binomial regression for premature (U75) CHD mortality count. Also included are the effects on premature CHD mortality count of a unit increase in the value of the explanatory variables.

Explanatory variable	*IRR*	*95% CI*	*p value*	*Effect of a 1% increase in explanatory variable on percentage change in premature mortality (CI).*	
**Percentage white patients**	1.008	(1.003, 1.012)	0.002	+0.8%	(0.3, 1.2)
**Deprivation score**	1.017	(1.011, 1.024)	<0.001	+1.7%	(1.1, 2.3)
**Prevalence of diabetes 2006/07**	1.114	(1.028, 1.208)	0.009	+11.4%	(2.8, 20.8)
**Percentage over 65**	1.059	(1.038, 1.081)	<0.001	+5.9%	(3.8, 8.1)
**Percentage male patients**	1.067	(1.038, 1.103)	<0.001	+6.7%	(3.8, 10.3)
**Number of GPs per 1000 patients**	1.197	(0.885, 1.619)	0.244	+19.7%	(-11.5, 61.9)
**Hypertension detection 2006/07**	0.978	(0.950, 1.007)	0.133	-2.2%	(-5.0, 0.7)
**% patients offered smoking cessation advice (SM02)**	1.002	(0.993, 1.011)	0.712	+0.2%	(-0.7, 1.1)
**% serum cholesterol (CHD08)**	0.991	(0.981, 1.000)	0.044	-0.9%	(-1.9, 0.0)
**% aspirin (CHD09)**	1.002	(0.982, 1.022)	0.884	+0.2%	(-1.8, 2.2)
**% of patients with recalled perception of being able to see preferred GP**	0.994	(0.989, 1.00)	0.036	-0.6%	(-1.1, 0.0)

*(one unit on scale for deprivation score).

Table 5 Impact on premature (U75) CHD mortality count of an improvement in primary care or decrease in population burden to the median from the upper or lower quartile as appropriate, given the current model.

Explanatory variable	*Description of change*	*Effect of improvement in primary care or decrease in population burden on percentage change in premature mortality (CI).*
**Percentage white patients**	*Decrease from upper quartile to median – decrease of 5.2% in percentage white patients.*	*-4.16% (-6.24%, -1.56%)*
**Deprivation score**	*Decrease from upper quartile to median – decrease of 10.8 units on scale.*	*-18.36% (-25.92%, -11.88%)*
**Prevalence of diabetes 2006/07**	*Decrease from upper quartile to median – decrease of 0.6% in diabetes prevalence.*	*-6.84% (-12.48%, -1.68%)*
**Percentage over 65**	*Decrease from upper quartile to median – decrease of 2.3% in percentage over 65.*	*-13.57% (-18.63%, -8.74%)*
**Percentage male patients**	*Decrease from upper quartile to median – decrease of 1.0% in percentage male patients.*	*-6.7% (-10.3%, -3.8%)*
**Number of GPs per 1000 patients**	*Increase from lower quartile to median – increase of 0.8 GPs per 1000 patients.*	*15.76% (-9.20%, 49.52%)*
**Hypertension detection 2006/07**	*Increase from lower quartile to median – increase of 2.3% in detection.*	*-5.06% (-11.27%, 1.61%)*
**% patients offered smoking cessation advice (SM02)**	*Increase from lower quartile to median – increase of 2.06% offered advice*	*0.41% (-1.44%, 2.27%)*
**% serum cholesterol (CHD08)**	*Increase from lower quartile to median – increase of 4.0% in achieving serum cholesterol target.*	*-3.6% (-7.6%, 0.0%)*
**% aspirin (CHD09)**	*Increase from lower quartile to median – increase of 2.0% in aspirin treatment.*	*0.4% (-3.6%, 4.4%)*
**% of patients with recalled perception of being able to see preferred GP**	*Increase from lower quartile to median – increase of 8.0% in patients recalling being able to see preferred GP.*	*-4.8% (-8.8%, -0.00%)*

For peer review only - http://bmjopen.bmj.com/site/about/guidelines.xhtml

Acknowledgements, Competing Interests and Funding

Competing Interests

All authors have completed the ICMJE uniform disclosure form at www.icmje.org/coi_disclosure.pdf and declare: KH had financial support from CLAHRC in the form of funding for PhD fees; no financial relationships with any organisations that might have an interest in the submitted work in the previous three years; no other relationships or activities that could appear to have influenced the submitted work.

Funding

The study formed part of the programme of the National Institute for Health Research (NIHR) Collaboration for Leadership in Applied Research and Care (CLARHC) in Leicestershire, Northamptonshire and Rutland. The CLARHC is funded by the NIHR. The views expressed in this paper do not necessarily reflect those of the NIHR. The funders had no role in study design, data collection and analysis, decision to publish, or preparation of the manuscript.

Contributor Statement

The study was conceived by KH, RB, JB and DJ. KH designed the study, carried out the analysis and drafted the initial manuscript. JB and DJ contributed to the statistical analysis. RB, JB and DJ contributed to drafting and editing the final manuscript and interpreting and reviewing the results of the statistical analysis.

Copyright Statement

The Corresponding Author has the right to grant on behalf of all authors and does grant on behalf of all authors, a worldwide licence (http://www.bmj.com/sites/default/files/BMJ%20Author%20Licence%20March%202013.doc) to the Publishers and its licensees in perpetuity, in all forms, formats and media (whether known now or created in the future), to i) publish, reproduce, distribute, display and store the Contribution, ii) translate the Contribution into other languages, create adaptations, reprints, include within collections and create summaries, extracts and/or, abstracts of the Contribution and convert or allow conversion into any format including without limitation audio, iii) create any other derivative work(s) based in whole or part on the on the Contribution, iv) to exploit all subsidiary rights to exploit all subsidiary rights that currently exist or as may exist in the future in the Contribution, v) the inclusion of electronic links from the Contribution to third party material where-ever it may be located; and, vi) licence any third party to do any or all of the above. All research articles will be made available on an Open Access basis (with authors being asked to pay an open access fee—see http://www.bmj.com/about-bmj/resources-authors/forms-policies-and-checklists/copyright-open-access-and-permission-reuse). The terms of such Open Access shall be governed by a Creative Commons licence—details as to which Creative Commons licence will apply to the research article are set out in our worldwide licence referred to above.

Ethical committee approval

Not required, as the study used only data that were already collected or publicly available, with no individuals or practices identified.

For peer review only

For peer review only

For peer review only - http://bmjopen.bmj.com/site/about/guidelines.xhtml

1 Department of Health. *Equity and excellence: Liberating the NHS*. London: Stationery Office, 2010. (White paper)

2 Department of Health. *The NHS Outcomes Framework 2013/14.* 2012. https://www.gov.uk/government/publications/nhs-outcomes-framework-2013-to-2014 (accessed 10 May 2013)

3 Townsend N, Wickramasinghe K, Bhatnagar P, *et al*. *Coronary heart disease statistics 2012 edition*. 2012. British Heart Foundation: London.

4 Murray CJ, Richards MA, Newton JN, *et al.* UK health performance: findings of the Global Burden of Disease Study 2010. *Lancet* 2013;**6736**. doi:10.1016/S0140-6736(13)60355-4

5 Macinko J, Starfield B, Shi L. Quantifying the health benefits of primary care physician supply in the United States. *Int J Health Serv* 2007;**37**:111–26.

6 Baker R, Sullivan E, Camosso-Stefinovic J, *et al.* Making use of mortality data to improve quality and safety in general practice: a review of current approaches. *Qual Saf Health Care* 2007;**16**:84–9.

7 Ashworth M, Schofield P, Doran T, *et al.* The Public Health Impact score: a new measure of public health effectiveness for general practices in England. *Br J Gen Pract* 2013;**63**:291–9.

8 Levene LS, Bankart J, Khunti K, *et al.* Association of primary care characteristics with variations in mortality rates in England: an observational study. *PLoS ONE* 2012;**7**(10):e47800. doi:10.1371/journal.pone.0047800

9 Baker R, Jones DR, Goldblatt P. Monitoring mortality rates in general practice after Shipman. *BMJ* 2003;**326**:274–6.

10 Kiran T, Hutchings A, Dhalla IA, *et al.* The association between quality of primary care, deprivation and cardiovascular outcomes: a cross-sectional study using data from the UK Quality and Outcomes Framework. *Epidemiol Community Health* 2010;**64**:927–34.

11 Levene LS, Baker R, Bankart MJG, *et al.* Association of features of primary health care with coronary heart disease mortality. *JAMA* 2010;**304**:2028–34.

12 Bottle A, Gnani S, Saxena S, *et al.* Association between quality of primary care and hospitalization for coronary heart disease in England: national cross-sectional study. *J Gen Intern Med* 2008;**23**:135–41.

13 Chauhan M, Bankart MJ, Labeit A, *et al.* Characteristics of general practices associated with numbers of elective admissions. *J Public Health* 2012;**34**:584–90.

For peer review only

14 Bankart MJG, Baker R, Rashid a, *et al.* Characteristics of general practices associated with emergency admission rates to hospital: a cross-sectional study. *Emerg Med J* 2011;**28**:558–63.

15 Rosenbaum PR, Rubin DB. Rates of Age-Adjusted Analyses Regression with Difficulties. *Biometrics* 1984;**40**:437–43.

16 Guo H-R. Age adjustment in ecological studies: using a study on arsenic ingestion and bladder cancer as an example. *BMC Public Health* 2011;**11**:820. doi:10.1186/1471-2458-11-820

17 Milyo J, Mellor JM. Income Inequality and Health Status in the United States Evidence from the Current Population Survey. *J Hum Resour* 2002;**37**:510–39.

18 Gardner W, Mulvey EP, Shaw EC. Regression analyses of counts and rates: Poisson, overdispersed Poisson, and negative binomial models. *Psychol Bull* 1995;**118**:392–404.

19 Freemantle N, Wood J, Griffin C, *et al.* What factors predict differences in infant and perinatal mortality in primary care trusts in England? A prognostic model. *BMJ* 2009;**339**:b2892–b2892.

20 Alter DA, Stukel TA, Newman A. The relationship between physician supply, cardiovascular health service use and cardiac disease burden in Ontario: Supply-need mismatch. *Can J Cardiol* 2008;**24**:187–93.

21 Bankart MJG, Baker R, Rashid a, *et al.* Characteristics of general practices associated with emergency admission rates to hospital: a cross-sectional study. *Emerg Med J* 2011;**28**:558–63.

22 Wheller L, Baker A, Griffiths C. Trends in premature mortality in England. *Health Stat Q* 2006;**31**:34-41

23 Allender S, Scarborough P, Keegan T, *et al.* Relative deprivation between neighbouring wards is predictive of coronary heart disease mortality after adjustment for absolute deprivation of wards. *J Epidemiol Community Health* 2012;**66**:803–8.

24 Lakhani A, Olearnik H, Eayres D. *Compendium of Clinicial and Helath Indicators: Data Definitions and User Guide for Computer File* 2011. The NHS Information Centre for health and social care. https://groups.ic.nhs.uk/archivedcompendia/public/Documents/June 2011/2011_06_User_Guide.pdf. (accessed 10 May 2013)

25 Quality and Outcomes Framework. *The Health and Social Care Information Centre: QOF online results database*. 2011 [online] Available at: http://www.qof.ic.nhs.uk [Accessed: 11 May 2013].

26 Dapartment of Health. *The GP Patient Survey*. 2007 [online] Available at: http://www.gp-patient.co.uk/download/Questionnaires/Y1 GP Patient Survey Questionnaire.pdf

27 Coleman T. Do financial incentives for delivering health promotion counselling work? Analysis of smoking cessation activities stimulated by the quality and outcomes. *BMC public health* 2010;**10**(167) doi:10.1186/1471-2458-10-1

For peer review only - http://bmjopen.bmj.com/site/about/guidelines.xhtml

28 Soljak M, Calderon-Larrañaga A, Sharma P, *et al.* Does higher quality primary health care reduce stroke admissions? *Br J Gen Pract* 2011;**61**(593):e801-7. doi:10.3399/bjgp11X613142.

29 Wolinsky FD, Bentler SE, Liu L, *et al.* Continuity of care with a primary care physician and mortality in older adults. *J Gerontol A Biol Sci Med Sci* 2010;**65**:421–8.

30 Downing A, Rudge G, Cheng Y, *et al.* Do the UK government's new Quality and Outcomes Framework (QOF) scores adequately measure primary care performance? A cross-sectional survey of routine healthcare data. *BMC Health Serv Res* 2007;**7**:166-172.

31 Soljak M, Samarasundera E, Indulkar T, *et al.* Variations in cardiovascular disease under-diagnosis in England: national cross-sectional spatial analysis. *BMC Cardiovasc Disord* 2011;**11**:12.

For peer review only

APPENDIX 1: Effect of choice of measure.

Table A1:1 Estimated incident rate ratios, associated 95% confidence intervals and associated *p* values for negative binomial regression for premature (U75) CHD mortality count.

	IRR	95% CI	*p* value
**Percentage white patients**	1.007	(1.003, 1.012)	0.002
**Deprivation score**	1.018	(1.012, 1.024)	<0.001
**Prevalence of diabetes 2006/07**	1.114	(1.029, 1.207)	0.008
**Percentage over 65**	1.051	(1.030, 1.072)	<0.001
**Number of GPs per 1000 patients**	1.173	(0.866, 1.589)	0.304
**Percentage male patients**	1.063	(1.028, 1.098)	<0.001
**Prevalence of hypertension 2006/07**	0.981	(0.953, 1.009)	0.182
**CHD quality achievement score (after Kiran et al)**	0.988	(0.978, 0.999)	0.037
**% of patients with recalled perception of being able to see preferred GP**	0.995	(0.989, 1.000)	0.061

APPENDIX 2: Effect of model choice

Table A2:1 Estimated regression coefficients for **weighted linear regression** showing the association between two CHD indicators and indirectly standardised **premature** (U75) CHD mortality rate.

	Coefficients	95% CI	*p* value
**Percentage white patients**	0.37	-0.14 to 0.88	0.152
**Deprivation score**	2.64	1.87 to 3.42	<0.001
**Prevalence of diabetes 2006/07**	3.12	-5.96 to 12.21	0.498
**List size**	-0.00015	-0.00055 to 0.00024	0.443
**Percentage over 65**	2.37	0.094 to 4.65	0.041
**Number of GPs per 1000 patients**	32.41	-3.13 to 67.95	0.074
**Percentage male patients**	2.86	-0.81 to 6.52	0.126
**Prevalence of hypertension 2006/07**	-2.65	-5.98 to 0.68	0.118
**% patients offered smoking cessation advice (SM02)**	0.22	-0.79 to 1.22	0.672
**% serum cholesterol (CHD08)**	-0.61	1.68 to 0.45	0.256
**% aspirin (CHD09)**	0.80	-1.33 to 2.94	0.461
**% of patients with recalled perception of being able to see preferred GP**	-0.77	-1.45 to -0.091	0.026
**R^2**	0.31		

Table A2: 2 Estimated regression coefficients for weighted linear regression showing the association between 'Kiran' CHD quality achievement score and indirectly standardised **premature** (U75) CHD mortality rate.

	Coefficients	95% CI	*p* value
**Percentage white patients**	0.36	-0.147 to 0.870	0.163
**Deprivation score**	2.72	1.96 to 3.47	<0.001
**Prevalence of diabetes 2006/07**	2.90	-6.18 to 11.98	0.529
**List size**	-0.000170	-0.000559 to 0.000220	0.392
**Percentage over 65**	2.19	-0.044 to 4.43	0.055
**Number of GPs per 1000 patients**	31.42	-4.07 to 66.92	0.082
**Percentage male patients**	2.67	-0.953 to 6.30	0.148
**Prevalence of hypertension 2006/07**	-240.1	-568.9 to 88.736	0.152
**CHD quality achievement score (after Kiran et al)**	-0.208	-1.45 to 1.03	0.742
**% of patients with recalled perception of being able to see preferred GP**	-0.781	-1.46 to -0.103	0.024
**R^2**	0.31		

For peer review only

APPENDIX 3: Comparison with all age mortality

Table A3:1 Estimated incident rate ratios and associated 95% confidence intervals and significance levels for negative binomial regression for two CHD indicators and all age CHD mortality rate.

	IRR	*95% CI*	*P value*
**Percentage white patients**	1.007	(1.003, 1.010)	<0.001
**Deprivation score**	1.013	(1.008, 1.017)	<0.001
**Prevalence of diabetes 2006/07**	1.089	(1.028, 1.153)	0.004
**Percentage over 65**	1.074	(1.059, 1.089)	<0.001
**Number of GPs per 1000 patients**	1.039	(0.830, 1.279)	0.725
**Percentage male patients**	1.008	(0.984, 1.033)	0.598
**Prevalence of hypertension 2006/07**	0.989	(0.969, 1.008)	0.249
**% patients offered smoking cessation advice (SM02)**	1.005	(0.999, 1.011)	0.117
**% serum cholesterol (CHD08)**	0.993	(0.986, 1.000)	0.036
**% aspirin (CHD09)**	0.995	(0.981, 1.009)	0.454
**% of patients with recalled perception of being able to see preferred GP**	0.998	(0.994, 1.002)	0.418

Table A3: 2 Estimated incident rate ratios and associated 95% confidence intervals and significance levels for negative binomial regression for 'Kiran' CHD quality achievement score and all age CHD mortality rate.

	**IRR**	**95% CI**	***p* Value**
**Percentage white patients**	1.006	(1.003, 1.010)	<0.001
**Deprivation score**	1.013	(1.009, 1.018)	<0.001
**Prevalence of diabetes 2006/07**	1.086	(1.024, 1.150)	0.005
**Percentage over 65**	1.074	(1.059, 1.089)	<0.001
**Number of GPs per 1000 patients**	1.022	(0.822, 1.271)	0.844
**Percentage male patients**	1.006	(0.982, 1.031)	0.639
**Prevalence of hypertension 2006/07**	0.383	(0.055, 2.681)	0.334
**CHD quality achievement score (after Kiran et al)**	0.992	(0.984, 0.999)	0.033
**% of patients with recalled perception of being able to see preferred GP**	0.999	(0.995, 1.003)	0.564

Table A3:3 Estimated regression coefficients for weighted linear regression showing the association between two CHD indicators and indirectly standardised all age CHD mortality rate.

	**Coefficients**	**95% CI**	***p* Value**
**Percentage white patients**	0.45	0.14 to 0.75	0.004
**Deprivation score**	1.33	0.87 to 1.80	<0.001
**Prevalence of diabetes 2006/07**	6.08	0.65 to 11.52	0.028
**List size**	0.000052	-0.00015 to 0.00025	0.610
**Percentage over 65**	0.269	-1.07 to 1.61	0.693
**Number of GPs per 1000 patients**	5.54	-16.65 to 27.72	0.623
**Percentage male patients**	-2.37	-4.63 to -0.114	0.040
**Prevalence of hypertension 2006/07**	-1.24	-3.25 to 0.77	0.225
**% patients offered smoking cessation advice (SM02)**	0.38	-0.21 to 0.97	0.205
**% serum cholesterol (CHD08)**	-0.34	-0.95 to 0.27	0.275
**% aspirin (CHD09)**	-0.80	-2.10 to 0.50	0.227
**% of patients with recalled perception of being able to see preferred GP**	-0.20	-0.57 to 0.17	0.284
R^2	0.32		

Table A3:4 Estimated regression coefficients for weighted linear regression showing the association between 'Kiran' CHD quality achievement score and indirectly standardised all age CHD mortality rate.

	**Coefficients**	**95% CI**	***p* Value**
**Percentage white patients**	0.44	0.13 to 0.75	0.005
**Deprivation score**	1.37	0.91 to 1.83	<0.001
**Prevalence of diabetes 2006/07**	6.25	0.79 to 11.71	0.025
**List size**	0.000034	-0.00017 to 0.00023	0.736
**Percentage over 65**	0.317	-1.01 to 1.64	0.638
**Number of GPs per 1000 patients**	5.44	-16.92 to 27.79	0.632
**Percentage male patients**	-2.71	-4.95 to -0.46	0.018
**Prevalence of hypertension 2006/07**	-119.79	-320.07 to 80.49	0.240
**CHD quality achievement score (after Kiran et al)**	-0.382	-1.12 to 0.360	0.311
**% of patients with recalled perception of being able to see preferred GP**	-0.178	-0.55 to 0.195	0.348
R^2	0.31		

For peer review only - http://bmjopen.bmj.com/site/about/guidelines.xhtml

For peer review only

STROBE 2007 (v4) Statement—Checklist of items that should be included in reports of *cross-sectional studies*

Section/Topic	Item #	Recommendation	Reported on page #
**Title and abstract**	1	(*a*) Indicate the study's design with a commonly used term in the title or the abstract	Title page
		(*b*) Provide in the abstract an informative and balanced summary of what was done and what was found	2
**Introduction**			
Background/rationale	2	Explain the scientific background and rationale for the investigation being reported	4 & 5
Objectives	3	State specific objectives, including any prespecified hypotheses	2 - abstract
**Methods**			
Study design	4	Present key elements of study design early in the paper	6
Setting	5	Describe the setting, locations, and relevant dates, including periods of recruitment, exposure, follow-up, and data collection	6
Participants	6	(*a*) Give the eligibility criteria, and the sources and methods of selection of participants	6
Variables	7	Clearly define all outcomes, exposures, predictors, potential confounders, and effect modifiers. Give diagnostic criteria, if applicable	6
Data sources/ measurement	8*	For each variable of interest, give sources of data and details of methods of assessment (measurement). Describe comparability of assessment methods if there is more than one group	6 & 7 & Table 2 p13
Bias	9	Describe any efforts to address potential sources of bias	*6 & 7*
Study size	10	Explain how the study size was arrived at	6
Quantitative variables	11	Explain how quantitative variables were handled in the analyses. If applicable, describe which groupings were chosen and why	6 & 7
Statistical methods	12	(*a*) Describe all statistical methods, including those used to control for confounding	7
		(*b*) Describe any methods used to examine subgroups and interactions	n/a
		(*c*) Explain how missing data were addressed	7
		(*d*) If applicable, describe analytical methods taking account of sampling strategy	n/a
		(*e*) Describe any sensitivity analyses	7 & 8
**Results**			

For peer review only

Participants	13*	(a) Report numbers of individuals at each stage of study—eg numbers potentially eligible, examined for eligibility, confirmed eligible, included in the study, completing follow-up, and analysed	6
		(b) Give reasons for non-participation at each stage	n/a
		(c) Consider use of a flow diagram	n/a
Descriptive data	14*	(a) Give characteristics of study participants (eg demographic, clinical, social) and information on exposures and potential confounders	Table 1 p13<br>Table 3 p14
		(b) Indicate number of participants with missing data for each variable of interest	n/a
Outcome data	15*	Report numbers of outcome events or summary measures	Table 3 p14
Main results	16	(*a*) Give unadjusted estimates and, if applicable, confounder-adjusted estimates and their precision (eg, 95% confidence interval). Make clear which confounders were adjusted for and why they were included	Table 1 p13<br>Table 3 p14
		(*b*) Report category boundaries when continuous variables were categorized	n/a
		(*c*) If relevant, consider translating estimates of relative risk into absolute risk for a meaningful time period	n/a
Other analyses	17	Report other analyses done—eg analyses of subgroups and interactions, and sensitivity analyses	7 & 8 & 9
**Discussion**			
Key results	18	Summarise key results with reference to study objectives	8 & 9
Limitations	19	Discuss limitations of the study, taking into account sources of potential bias or imprecision. Discuss both direction and magnitude of any potential bias	10
Interpretation	20	Give a cautious overall interpretation of results considering objectives, limitations, multiplicity of analyses, results from similar studies, and other relevant evidence	9 & 11
Generalisability	21	Discuss the generalisability (external validity) of the study results	11
**Other information**			
Funding	22	Give the source of funding and the role of the funders for the present study and, if applicable, for the original study on which the present article is based	16

*Give information separately for cases and controls in case-control studies and, if applicable, for exposed and unexposed groups in cohort and cross-sectional studies.

Note: An Explanation and Elaboration article discusses each checklist item and gives methodological background and published examples of transparent reporting. The STROBE checklist is best used in conjunction with this article (freely available on the Web sites of PLoS Medicine at http://www.plosmedicine.org/, Annals of Internal Medicine at http://www.annals.org/, and Epidemiology at http://www.epidem.com/). Information on the STROBE Initiative is available at www.strobe-statement.org.

For peer review only

Modelling factors in primary care quality improvement: a cross-sectional study of premature CHD mortality.

Journal:	*BMJ Open*
Manuscript ID:	bmjopen-2013-003391.R1
Article Type:	Research
Date Submitted by the Author:	09-Sep-2013
Complete List of Authors:	Honeyford, Kate; University of Leicester, Health Sciences<br>Baker, Richard; University of Leicester, Health Sciences<br>Bankart, M. John; Keele University, Institute of Primary Care<br>Jones, David; University Of Leicester, Health Sciences
**Primary Subject Heading**:	Public health
Secondary Subject Heading:	Health services research, Epidemiology, Cardiovascular medicine
Keywords:	PRIMARY CARE, STATISTICS & RESEARCH METHODS, Cardiac Epidemiology < CARDIOLOGY, Coronary heart disease < CARDIOLOGY

SCHOLARONE™
Manuscripts

TITLE PAGE

Title: Modelling factors in primary care quality improvement: a cross sectional study of premature CHD mortality.

Corresponding author:

Kate Honeyford MSc

Adrian Building,

University Road,

Leicester

LE1 7RH

ceh28@le.ac.uk

Tel: 0116 229 7254/7255

Fax: 0116 229 7250

Co-authors:

Professor Richard Baker, Dept of Health Sciences, University of Leicester, Leicester, UK.

Dr M. John G. Bankart, Insitute of Primary Care and Health Sciences, Keele University, Keele, UK.

Professor David Jones, Dept Health Sciences, University of Leicester, Leicester, UK.

Key words:

Primary health care

Regression analysis

Mortality, premature

Coronary disease

Cross-sectional studies

Word count: 3995 (including section headings)

For peer review only

For peer review only - http://bmjopen.bmj.com/site/about/guidelines.xhtml

ABSTRACT

Objectives

i) To identify features of primary care quality improvement associated with improved health outcomes using premature coronary heart disease (CHD) mortality as an example, and

ii) to determine impacts of different modelling approaches.

Design

Cross sectional study of mortality rates in 229 general practices.

Setting

General practices from three East Midlands primary care trusts.

Participants

Patients registered to practices above between April 2006 and March 2009.

Main outcome measures

Numbers of CHD deaths in those aged under 75 (premature mortality) and at all ages in each practice.

Results

Both population characteristics and markers of quality of primary care were associated with variations in premature CHD mortality. Increasing levels of deprivation percentages of practice populations on practice diabetes registers, white, over 65 and male were all associated with increasing levels of premature CHD mortality. Control of serum cholesterol levels in those with CHD and the percentage of patients recalling access to their preferred GP were both associated with decreased levels of premature CHD mortality. Similar results were found for all-age mortality. A combined measure of quality of primary care for CHD comprising 12 QOF indicators was associated with decreases in both all-age and premature CHD mortality. The selected models suggest that practices in less deprived areas may have up to 20% lower premature CHD mortality than those with median deprivation and that improvement in the CHD care quality from 83% (lower quartile) to 86% (median) could reduce premature CHD mortality by 3.6%. Different modelling approaches yielded qualitatively similar results.

Conclusions

High quality primary care, including aspects of access to and continuity of care, detection and management, appears to be associated with reducing CHD mortality. The impact on premature CHD mortality is greater than on all-age CHD mortality. Determining the most useful measures of quality of primary care needs further consideration.

For peer review only

For peer review only - http://bmjopen.bmj.com/site/about/guidelines.xhtml

Article Focus:

- CHD is a major cause of premature deaths in the UK; primary care can play a role in modifying risk factors in populations.
- This study identifies features of primary care associated with lower levels of premature coronary heart disease.
- A variety of methods have been used to model associations; this paper explores the impact of different modelling approaches.

Key Messages:

- High quality primary care, as measured through QOF clinical indicators and relational continuity, are associated with lower levels of premature CHD mortality.
- To inform future monitoring and development of primary care, more reliable data relating to smoking and other lifestyle factors is necessary.
- Results and their interpretation show some sensitivity to modelling approaches.

Strengths and weaknesses

- The associations between several modifiable risk factors and premature CHD mortality are considered, and compared to those for all age CHD mortality.
- The study examines the impact of model choice and the measure of primary care quality on results and interpretation.
- The lack of reliable data relating to lifestyle factors such as smoking and obesity rates mean that important covariates are not included in the model.

For peer review only

For peer review only - http://bmjopen.bmj.com/site/about/guidelines.xhtml

INTRODUCTION

In England, in common with health systems in many other countries, health outcomes are increasingly being used to plan and manage health services.[1] A framework for health outcomes has been introduced to provide an overview of how the national health service (NHS) is performing and as a catalyst for driving quality.[2] One of the framework's five domains is the prevention of premature death, one of the twelve indicators in this domain being death from cardiovascular disease under the age of 75 years.

Whilst mortality caused by coronary heart disease (CHD) has been falling steadily since the 1980s[3] it is still the leading cause of years of life lost in the UK. The Global Burden Disease Study[4] shows that CHD mortality rates in the UK are significantly above the mean of those in comparator countries. Although population characteristics including lifestyle (eg obesity, smoking) and hypertension are known to be associated with CHD mortality, primary care may have a role in reducing CHD mortality. For example, in the US, greater supply of primary care physicians is associated with lower heart disease mortality,[5] although there is little evidence of a similar association in the UK.[6] Since general practices serve lists of registered patients, there is potential to relate mortality in practice populations to both population characteristics and performance of practices, and thereby assist practices in planning and monitoring their activities to reduce population mortality.[7] A conceptual model describing how the delivery of primary care can modify the impact of population based characteristics on health outcomes has been proposed.[8] It includes interventions which target both the morbid and healthy populations via early detection, prevention and appropriate management of people with established disease and highlights the importance of access and continuity of care. However, the size of practice populations and the small numbers of deaths present methodological difficulties.[9] In seeking to understand how primary care performance affects mortality in practice populations, consideration must be given to **(1)** the measurement of the performance of primary care; and **(2)** selection of the underlying statistical model. In recent years, information about the performance of general practices has become available, in addition to other practice characteristics such as numbers of registered patients and numbers of general practitioners. The Quality Outcomes Framework (QOF) has provided data in four domains (clinical; organisational; patient experience and additional). Within the clinical domain are several chronic conditions (including CHD), each made up of individual indicators. For each indicator points are awarded to practices based on the percentage of patients for whom the target has been achieved (known as the underlying achievement). Score and underlying achievement for each indicator are publicly available. Additionally, QOF contains 'prevalence registers' showing the percentage of the practice population identified by the practice as having a particular condition or disease.

In investigating aspects of practice performance associated with various outcomes, Kiran et al used combinations of individual indicators' underlying achievement to give a devised 'CHD quality achievement score'.[10]
QOF prevalence registers have also been used as measures of quality of primary care; for example greater numbers of people on practice hypertension registers in primary care trusts (PCTs) have been shown to be associated with lower CHD mortality, indicating that improved detection of hypertension has a positive impact on outcome.[8 11]

For peer review only - http://bmjopen.bmj.com/site/about/guidelines.xhtml

Differences between study results may be partly explained by different usage of QOF data. For example, Kiran et al [10] found that the overall CHD quality achievement score was negatively associated with CHD mortality. However, other studies have shown no association between *individual* indicators and CHD mortality [8] and CHD admissions [12] or overall scores and emergency and elective admissions. [13 14]

Statistical Method and Model Selection

Different statistical models may be used. A common method is multiple linear regression with age-standardised mortality rates as the dependent variable. However, since in research of this type age-adjusted variables are rarely available multiple linear regression of standardised mortality rates can lead to biased risk estimates.[15–17]

Poisson models have the advantage of considering deaths as count data; age and sex can then be included in the model as explanatory variables, thus overcoming the lack of age standardised explanatory variables. As the counts are often over-dispersed, a negative binomial model may be more appropriate than a Poisson model.[18] Models based on count data, for example the numbers of deaths or hospital admissions, can also be found in the literature.[8 13 19–21]

Premature Mortality

This study defines premature mortality in those under 75, in line with the Outcomes Framework and Office of National Statistics preferences.[22] Few studies have considered the associations between quality of primary health care, as measured by QOF, and *premature* mortality. Allender et al[23] demonstrated a stronger association between socio-economic deprivation and premature CHD mortality, compared to all-age CHD mortality. In a study of CHD hospitalisation and primary care[12] patients were divided into 45-74 and 75 and over; socio-economic status was found to be more important in the younger age group, but no difference was found in the association of quality of care and health outcomes in the two different age groups.

In this study, we investigate the choice of performance indicators and statistical models to explain premature and all-age practice population mortality from CHD. The overall aim is to identify key aspects of primary care quality improvement having potential to improve health outcomes, here to reduce premature CHD mortality.

For peer review only - http://bmjopen.bmj.com/site/about/guidelines.xhtml

METHODS

This is an observational study of CHD mortality between April 2006 and March 2009 in 229 practices in the East Midlands. All 230 practices that were open for the three years of the study were eligible for inclusion. One practice offering a service to a restricted patient group was excluded from the study. The total population covered by the study is just under 1.7 million people. In this study premature CHD mortality will be the main outcome measure; this will be compared with all-age CHD mortality. Counts of deaths will be modelled using negative binomial regression. Results will be compared to those from weighted linear regression of indirectly standardised mortality ratios to determine the impact of the model adopted on the overall findings and conclusions of the analysis.

CHD mortality counts for each practice were constructed from the Primary Care Mortality Database for the period, supplied by the relevant PCTs. Each record included date of birth, date of death, underlying cause of death and the general practice code for the patient's practice. Each death was linked to a general practice using these codes. Deaths with underlying cause identified as CHD (ICD-10 codes: I20-I25) were included in the study. In addition, indirectly standardised mortality rates (SMR) were calculated for each practice, based on England and Wales' standard rates and midyear estimates of practice populations provided by PCTs in line with NCHOD's methodology.[24] Since numbers of deaths in each practice per year were low, both counts and SMRs were aggregated over the three years.

QOF clinical data are publicly available for all practices in the sample for 2006/07, 2007/08 and 2008/09.[25] The 2006/7 indicators were used since the primary care described by indicators for that year will have had an impact on the largest proportion of patients included in the analysis. Levene et al[8] classify primary healthcare into prevention, early detection and appropriate management. QOF indicators can be classified following this approach:

Prevention: Prevention of smoking and obesity. Reducing the number of people who are smoking or are obese would be useful indicators of prevention; however neither of these is available for practice populations. The QOF indicator which details the percentage of patients having a range of conditions who have been offered smoking cessation advice is being used as a measure of prevention of smoking (SM02).

Early detection: Detection of hypertension. Levene et al [8 11] argue that given only a little more than half of the people found to have hypertension in population surveys in England are included on QOF hypertension registers these can be used as a useful measure of hypertension detection.

Appropriate disease management: Indicators identified by Levene et al have been selected for this analysis:- **(a)** the control of serum cholesterol in patients on practice CHD registers (CHD08) and **(b)** the percentage of the practice CHD register who are being treated with aspirin (or equivalent) (CHD09).

See Table 1 for more detailed descriptions of QOF indicators.

For peer review only - http://bmjopen.bmj.com/site/about/guidelines.xhtml

The CHD quality achievement score devised by Kiran et al[10] is included in a comparative analysis. The measure is the mean underlying achievement of 12 indicators (see Table 1).

Access and sustained relationships: one question from the General Practice Patient Survey[26] relating to patients' recall of being able to consult a particular GP has been selected as an indicator of the ability to access care and whether patients prefer to have access to a sustained relationship in line with the work of Levene et al.[8] In addition, the number of GPs per 1000 patients is being included as a measure of GP supply.

Characteristics of the **practice population** have been selected based on previous research, the use of count data and availability. The percentage of the practice population who are male and are aged over 65 are included because they are known to be associated with higher rates of CHD mortality. List size is included as a measure of exposure in the negative binomial model, to take into variability in practice size and therefore the number at risk varies from practice to practice. List size is included in multiple regression for consistency. Diabetes prevalence, ethnicity and deprivation are included as previous research has shown these to be important in explaining variation in CHD mortality ([8 11], [10] and [8 10] respectively). Neither obesity nor smoking rates are included as the MSOA measures available are unlikely to describe rates within practice populations accurately. The sources of information used for each of these are summarised in Table 2. Since CHD prevalence is highly correlated with both age and hypertension detection it was not included in the model to avoid collinearrity problems.

Eight practices have missing data for the number of GPs (fte); three practices have missing data from the GP Patient survey. The main analysis was repeated using a range of values; choice of value was not important. Either the median value or values from following years have been used.

Statistical analysis

Counts of deaths in each practice are modelled here using negative binomial regression in preference to Poisson regression since the data are over-dispersed (over-dispersion parameter $\alpha=0.023$, $p<0.05$). To explore how sensitive the results and interpretation are to the modelling approach used, ordinary least squares (OLS) linear regression with the standardised mortality ratio (SMR) as the dependent variable was also carried out, using the inverse of the variance of SMR as a weighting to account for uncertainty in the standardised mortality rates, in line with the work of Kiran et al.[10]

For peer review only - http://bmjopen.bmj.com/site/about/guidelines.xhtml

RESULTS

Table 3 shows summary data of counts of death, mortality rates, and characteristics of both practice populations and primary care for the 229 practices, serving approximately 1.7 million patients in 2006/07. Table 4 shows the estimates of incident rate ratios (IRRs) in the presented negative binomial model, including 95% confidence intervals and *p* values for all explanatory variables; the impact of a one unit increase in each explanatory variable is also included to aid the interpretation of IRRs. As in many models in this field reporting pseudo R^2, adjusted pseudo R^2 is low, although the predicted and observed counts are relatively close (Lin's concordance coefficient : 0.86). There is no pattern in the residuals and removal of identified outliers did not materially affect interpretation of the model.

Table 5 shows the potential impact that changes in population or service characteristics could have on premature CHD mortality, given the current model. For each explanatory variable the change from either the upper or lower quartile to the median is used to show how decreases in the 'risk' in the practice population or increases in the quality of primary care could impact on mortality, if the model is adequate and relationships causal.

Sensitivity analysis explored the impact of various smoking and obesity indicators. Inclusion of QOF registers or modelled estimates for local area obesity prevalence do not materially change the coefficients; there is no evidence that the model is improved by so doing. Similar results are found when both modelled estimates of smoking rates and QOF indicators relating to smoking are included. None of these indicators has been included in the final model.

Population Characteristics

The main predictors of variation in premature CHD mortality of the population characteristics included in the model were deprivation, age (percentage aged 65 and over), percentage on the diabetes register and percentage male. Table 5 suggests that practices in less deprived areas may have up to 20% lower premature CHD mortality counts than those with deprivation at the median.

Service Characteristics

Increases in levels of service in terms of the percentage of the CHD register whose serum cholesterol is controlled (CHD08), and the percentage of patients who recalled being able to see their preferred GP, were associated with lower levels of premature CHD mortality, as was (less clearly) increased hypertension detection (as measured through QOF hypertension prevalence registers).

Similarly, there is a suggestion that increases in GPs per 1000 patients and the percentage of the CHD register being treated with aspirin (or an alternative) are both associated with higher premature mortality. Table 5 allows interpretation of these key variables in the current model, and suggests that increasing the percentage of patients who recall being able to see their preferred GP from a lower performing practice (typically 80%) to a median performing practice (88%) may reduce premature CHD mortality counts by 4.8%. Similarly, increasing the percentage of patients on the CHD register for whom

For peer review only - http://bmjopen.bmj.com/site/about/guidelines.xhtml

their serum cholesterol is below 5mmol/l from a low performing practice (78%) to a median performing practice (82%) may reduce premature CHD mortality counts by 3.6%.

Effect of choice of measure of quality of primary care

Similar results are found when the Kiran CHD quality achievement score is used in the model instead of the two separate CHD quality indicators. In the subsequent model an improvement from 83% (lower quartile) mean achievement in the Kiran overall CHD achievement score to 86% (median) reduces CHD mortality counts by 3.6% (see Appendix 1 for more information).

Effect of model selection

When a weighted multiple linear regression model for standardised mortality ratio was used, the directions of associations were the same as those described above. However, the confidence intervals for the β-coefficients for both prevalence of diabetes and the CHD quality achievement measures now include zero, and hence interpretation of the results might be less clear cut (see Appendix 2 for more information).

Comparison with all age mortality

The results for all age CHD mortality are broadly similar to those for premature CHD mortality. Increases in white ethnicity, deprivation and diabetes prevalence and an increase in GPs per 1000 patients are associated with increases in mortality, whereas improvements in the remaining service characteristics correspond to decreases in mortality counts. However, the confidence intervals for the IRRs for all service characteristics include one, impacting on the interpretation of the importance of these variables.

DISCUSSION

Statement of principal findings

Both population characteristics and the quality of primary care were found to be associated with variations in premature CHD mortality. Increasing levels of deprivation, the percentage of the practice population who were on the practice diabetes register, who were white, over 65 and who were male were all associated with increasing levels of premature CHD mortality. Control of serum cholesterol levels in those with CHD and the percentage of patients who could recall being able to see their preferred GP were both associated with decreased levels of premature CHD mortality. Similar results were found when all age mortality was considered. The combined measure of the quality of primary care was associated with a decrease in both all-age and premature CHD mortality. However, it is difficult to determine which individual indicators within this measure are key to reducing CHD mortality. The evidence that hypertension detection is associated with decreased CHD mortality is less clear than has been found by Levene et al at PCT level.[8] Different modelling approaches yielded qualitatively similar

For peer review only - http://bmjopen.bmj.com/site/about/guidelines.xhtml

results; however, detailed interpretation of the results would be model dependent, particularly if statistical significance were rigidly applied as a criterion of importance.

Strengths and weaknesses of this study

A key strength of this study is that it considers the association of features of primary care with *premature* CHD mortality, and whilst the overall relationships are similar to those found when all age mortality is considered, some associations are stronger, for example, that with patients' recall of being able to see their preferred GP.

Most explanatory variables in this study describe the adult practice population and not those under 75. To develop our understanding of the different contributions made by different variables in explaining premature mortality it would be helpful to have reliable data describing different age groups within practice populations.

This study allows useful consideration of the impact of the measure of primary care quality used. Whilst the higher underlying achievement in overall CHD achievement score devised by Kiran is associated with decreased premature mortality, this doesn't allow policy makers or clinicians to determine which of the 12 indicators are most important. The examination of the individual indicators shows that increasing the percentage of patients on the CHD register whose serum cholesterol is below 5mmol/l is associated with decreasing mortality, but it is unclear whether this is the most important indicator. Unfortunately, as patient level data relating to QOF indicators are not available for this study, it is not possible to determine whether combinations of indicators within individuals are key or if there are interactions between indicators and other characteristics of individuals, for example ethnicity or gender.

Reliable information about smoking rates, alcohol consumption and obesity within practice populations is lacking, and having access to these data would be likely to improve the model fit. Whilst various estimates are available, many of these are modelled estimates for geographical areas, not practice populations, based on levels of deprivation, ethnicity and age, which are anyway included in the models in this study. Integrated Household Study smoking data is available at local area level, and could be used to estimate smoking prevalence in practice populations. However, it was not possible to match these to practice populations for this study. The introduction of new smoking indicators in the 2012/13 QOF asks practices to record the smoking status of those aged 15 years and over and to record an offer of support and treatment to those who smoke, which may start to give a fuller picture of smoking prevalence in practice populations. Whilst a QOF smoking indicator relating to smoking cessation advice has been included in this study as a measure of prevention, the wording asks only for 'an offer of support and treatment' which may throw doubt on its validity.[27] White ethnicity percentage was included in the model; it is highly correlated (R_p: -0.993) with the percentage who are Asian. More refined ethnicity information was not available for thie study.

This study allows the impact of statistical method and model selection to be considered. Whilst the directions of associations are generally not affected by the choice of model the statistical significance of

For peer review only - http://bmjopen.bmj.com/site/about/guidelines.xhtml

the results vary between models for several key variables. For example, neither the prevalence of diabetes nor the CHD quality achievement score would be considered to be significantly associated with premature mortality using a weighted linear regression model for SMRs.

Relation to other studies

Previous research has shown that primary care is important in improving health outcomes but that the precise aspects of primary care which are most important are not clear.[8 10 13 14 28] This study confirms that position, but explores other aspects of the problem.

An association between continuity of care and reduced mortality in older patients has been found in the US[29] and Bankart et al[14] have shown that in practices with higher mean rates of satisfaction with being able to consult a preferred GP, emergency admissions are lower. These findings give further support to the importance of continuity of care in improving health outcomes.
Levene et al have found that hypertension detection was associated with reduced levels of CHD mortality in two studies completed at PCT level. In their Global Burden of Disease Study 2010 Murray et al[4] highlight the importance of early detection and long term management of high blood pressure as a 'clear route to accelerate progress for the leading causes of avoidable cardiovascular mortality'. However, the evidence of the importance of hypertension detection in this study is not clear cut. It may be that QOF prevalence registers are not a useful measure of detection for individual practices. Low QOF prevalence rates may be due to lower levels of hypertension rather than lower detection and conversely high QOF prevalence rates may be due to higher underlying prevalence rather than improved detection. More work to identify the most useful combination of QOF indicators to summarise hypertension detection is necessary.

Consistent with other studies, socio-economic deprivation is the main predictor of CHD mortality. Both Bottle[12] and Allender[23] have found that socio-economic deprivation is more strongly associated with mortality in younger age groups and this study has confirmed this pattern.

Whilst some studies clearly state the reasons for their selection of statistical modelling approach and/or potential explanatory variables (for example [13 14 19 30 31]), many studies do not. Here we find some indication that qualitative interpretations of results are robust to model choice. However, more detailed interpretation of results is likely to vary between models, particularly if there is undue reliance on simplistic interpretation of statistical significance.

Meaning of the study

This study adds to the body of research demonstrating that high quality primary care is associated with improving health outcomes. Aspects of both continuity of care and disease management have been identified as having a bigger impact on reducing premature CHD mortality than all-age CHD mortality. Whilst the most important individual indicators relating to disease management have not been identified, there is clear evidence that improving achievement in QOF indicators is associated with

For peer review only - http://bmjopen.bmj.com/site/about/guidelines.xhtml

decreasing CHD mortality. The importance of continuity of care, again shown here, strongly suggests that this is an area for general practices to prioritise. The findings also suggest that data about outcomes such as premature CHD mortality could be used by practices to monitor and, over several years, plan their care to improve population health.

The ongoing importance of socio-economic deprivation in explaining higher levels of mortality cannot be ignored. Understanding the relationship between deprivation and health outcomes more precisely remains an important area of further study.

Unanswered questions and future research

The lack of reliable practice level information on key areas such as obesity, alcohol and smoking prevalence has an important impact on primary care research and is an important health information issue needing effective attention as the NHS undergoes major changes. Whilst QOF indicators relating to smoking prevalence have been introduced, the reliability of these measures will need to be scrutinised. To further our understanding of the relative importance of different QOF indicators it would be useful to study individual QOF indicators at patient level and how they interact with each other and with characteristics of individuals on an individual patient basis. In future studies, the impact of the introduction of health checks in 2009 should be explored.

CONCLUSION

Improving the quality of primary care will play an important part in decreasing premature mortality, and there is evidence that high underlying achievements in QOF clinical indicators are a useful measure of quality primary care. Continuity of care, in a country with universal access to healthcare, is important and should not be underestimated by policy makers and clinicians. Nonetheless lifestyle factors are also important, but our ability to study them adequately in primary care, or to evaluate the role of primary care in addressing them, is currently limited by the quality of measures at practice level. If primary care services, delivered by clinical commissioning groups are to be monitored and developed using the new NHS Outcomes Framework, better data and careful modelling and interpretation are vital.

For peer review only - http://bmjopen.bmj.com/site/about/guidelines.xhtml

Tables

Table 1: QOF indicators used to compile the overall CHD quality achievement score.

Clinical domain			Summary statistics in this study (median and interquartile range)
**CHD**	CHD06	The percentage of patients with CHD in whom the last blood pressure reading (measured in the previous 15 months) is ≤150/90 mm Hg.	90 (85, 93)
	CHD08	The percentage of patients with CHD whose last measured total cholesterol level (measured in the previous 15 months) is ≤5 mmol/l.	82 (78, 87)
	CHD09	The percentage of patients with CHD with a record in the previous 15 months that aspirin, an alternative antiplatelet therapy, or an anti-coagulant is being taken (unless a contraindication or adverse effects are recorded).	95 (93, 97)
	CHD10	The percentage of patients with CHD who are currently treated with a β-blocker (unless a contraindication or adverse effects are recorded).	73 (66, 81)
	CHD11	The percentage of patients with a history of myocardial infarction (diagnosed after 1 April 2003) who are currently treated with an angiotensin-converting enzyme inhibitor or angiotensin II antagonist.	92 (88, 97)
**Stroke and TIA**	STROKE06	The percentage of patients with TIA or stroke in whom the last blood pressure reading (measured in the previous 15 months) is ≤150/90 mm Hg.	88 (82, 92)
	STROKE08	The percentage of patients with TIA or stroke whose last measured total cholesterol level (measured in the previous 15 months) is ≤5 mmol/l.	76 (70, 83)
**Hypertension**	BP05	The percentage of patients with hypertension in whom the last blood pressure reading (measured in the previous 9 months) is ≤150/90 mm Hg.	77 (73, 83)
**Diabetes mellitus**	DM12	The percentage of patients with diabetes in whom the last blood pressure reading is ≤145/85 mm Hg.	80 (74, 85)
	DM17	The percentage of patients with diabetes whose last measured total cholesterol level (measured in the previous 15 months) is ≤5 mmol/l.	83 (77, 87)
	DM20	The percentage of patients with diabetes in whom the last HbA1C is ≤ 7.5 (or equivalent test/reference range depending on laboratory) in the previous 15 months.	67 (61, 73)
**Smoking**	SMOKE02	The percentage of patients with any or any combination of the following condition: CHD, stroke or TIA, hypertension, diabetes, COPD or asthma, who smoke and whose notes contain a record that smoking cessation advice or referral to a specialist service, where available, has been offered within the previous 15 months.	94 (92, 96)
**Kiran CHD overall achievement score**		Mean of the above 12 indicators	83 (80, 86)

Table 2: Characteristics of practice populations.

Measure	Source of data
**Deprivation indices**	Based on Index of Multiple Deprivation 2007.
**% of GP list on diabetes register**	QOF prevalence register 2006/07.
**% White ethnicity**	Based on hospital episode statistics.
**% of population who are over 65**	Based on mid-year practice population estimates provided by the PCT.
**% of population who are male**	Based on mid-year practice population estimates provided by the PCT.
**List size**	Based on mid-year practice population estimates provided by the PCT.

For peer review only - http://bmjopen.bmj.com/site/about/guidelines.xhtml

Table 3 Summary statistics for mortality counts, population characteristics and service characteristics.

	Summary statistics in this study Median (IQR)
**Mortality data**	
Indirectly Standardised CHD mortality	95.9 (78.3, 119.9)
Indirectly Standardised CHD mortality – under 75	92.4 (64.0, 135.6)
Crude rate per 1000	1.30 (0.95, 1.57)
Crude rate per 1000 – under 75	0.43 (0.28, 0.58)
**Practice characteristics**	
Deprivation indices	16.2 (10.0, 27.0)
% of GP list on diabetes register	3.8 (3.3, 4.4)
% White ethnicity	89.9 (77.5, 94.1)
% of population who are over 65	14.7 (12.1, 17.0)
% of population who are male	50.2 (49.5, 51.2)
List size	6435 (3749, 10319)
**Service characteristics**	
GPs per 1000	0.55 (0.47, 0.64)
% patients with recalled perception of being able to see preferred GP	88 (80, 93)
% of GP registered list on hypertension register	12.3 (11.0, 14.7)
The percentage of patients with any or any combination of the following conditions: coronary heart disease, stroke or TIA, hypertension, diabetes, COPD or asthma who smoke whose notes contain a record that smoking cessation advice or referral to a specialist service, where available, has been offered within the previous 15 months. SM02	94 (92, 96)
The percentage of patients with CHD whose last measured total cholesterol level (measured in the previous 15 months) is ≤5 mmol/l. CHD08	82 (78, 87)
The percentage of patients with CHD with a record in the previous 15 months that aspirin, an alternative antiplatelet therapy, or an anti-coagulant is being taken (unless a contraindication or adverse effects are recorded). CHD09	95 (93, 97)
CHD overall achievement score[10]	83 (80, 86)

For peer review only - http://bmjopen.bmj.com/site/about/guidelines.xhtml

Table 4 Estimated incident rate ratios, associated 95% confidence intervals and associated *p* values for negative binomial regression for premature (U75) CHD mortality count. Also included are the effects on premature CHD mortality count of a unit increase in the value of the explanatory variables.

Explanatory variable	*IRR*	*95% CI*	*p value*	*Effect of a 1% increase in explanatory variable on percentage change in premature mortality (CI).*	
**Percentage white patients**	1.008	(1.003, 1.012)	0.002	+0.8%	(0.3, 1.2)
**Deprivation score**	1.017	(1.011, 1.024)	<0.001	+1.7%	(1.1, 2.3)
**Prevalence of diabetes 2006/07**	1.114	(1.028, 1.208)	0.009	+11.4%	(2.8, 20.8)
**Percentage over 65**	1.059	(1.038, 1.081)	<0.001	+5.9%	(3.8, 8.1)
**Percentage male patients**	1.067	(1.038, 1.103)	<0.001	+6.7%	(3.8, 10.3)
**Number of GPs per 1000 patients**	1.197	(0.885, 1.619)	0.244	+19.7%	(-11.5, 61.9)
**Hypertension detection 2006/07**	0.978	(0.950, 1.007)	0.133	-2.2%	(-5.0, 0.7)
**% patients offered smoking cessation advice (SM02)**	1.002	(0.993, 1.011)	0.712	+0.2%	(-0.7, 1.1)
**% serum cholesterol (CHD08)**	0.991	(0.981, 1.000)	0.044	-0.9%	(-1.9, 0.0)
**% aspirin (CHD09)**	1.002	(0.982, 1.022)	0.884	+0.2%	(-1.8, 2.2)
**% of patients with recalled perception of being able to see preferred GP**	0.994	(0.989, 1.00)	0.036	-0.6%	(-1.1, 0.0)

*(one unit on scale for deprivation score).

Table 5 Impact on premature (U75) CHD mortality count of an improvement in primary care or decrease in population burden to the median from the upper or lower quartile as appropriate, given the current model.

Explanatory variable	*Description of change*	*Effect of improvement in primary care or decrease in population burden on percentage change in premature mortality (CI).*
**Percentage white patients**	***Decrease from upper quartile to median – decrease of 5.2% in percentage white patients.***	***-4.16% (-6.24%, -1.56%)***
**Deprivation score**	***Decrease from upper quartile to median – decrease of 10.8 units on scale.***	***-18.36% (-25.92%, -11.88%)***
**Prevalence of diabetes 2006/07**	***Decrease from upper quartile to median – decrease of 0.6% in diabetes prevalence.***	***-6.84% (-12.48%, -1.68%)***
**Percentage over 65**	***Decrease from upper quartile to median – decrease of 2.3% in percentage over 65.***	***-13.57% (-18.63%, -8.74%)***
**Percentage male patients**	***Decrease from upper quartile to median – decrease of 1.0% in percentage male patients.***	***-6.7% (-10.3%, -3.8%)***
**Number of GPs per 1000 patients**	***Increase from lower quartile to median – increase of 0.8 GPs per 1000 patients.***	***15.76% (-9.20%, 49.52%)***
**Hypertension detection 2006/07**	***Increase from lower quartile to median – increase of 2.3% in detection.***	***-5.06% (-11.27%, 1.61%)***
**% patients offered smoking cessation advice (SM02)**	***Increase from lower quartile to median – increase of 2.06% offered advice***	***0.41% (-1.44%, 2.27%)***
**% serum cholesterol (CHD08)**	***Increase from lower quartile to median – increase of 4.0% in achieving serum cholesterol target.***	***-3.6% (-7.6%, 0.0%)***
**% aspirin (CHD09)**	***Increase from lower quartile to median – increase of 2.0% in aspirin treatment.***	***0.4% (-3.6%, 4.4%)***
**% of patients with recalled perception of being able to see preferred GP**	***Increase from lower quartile to median – increase of 8.0% in patients recalling being able to see preferred GP.***	***-4.8% (-8.8%, -0.00%)***

For peer review only - http://bmjopen.bmj.com/site/about/guidelines.xhtml

Acknowledgements, Competing Interests and Funding

Competing Interests

All authors have completed the ICMJE uniform disclosure form at www.icmje.org/coi_disclosure.pdf and declare: KH had financial support from CLAHRC in the form of funding for PhD fees; no financial relationships with any organisations that might have an interest in the submitted work in the previous three years; no other relationships or activities that could appear to have influenced the submitted work.

Funding

The study formed part of the programme of the National Institute for Health Research (NIHR) Collaboration for Leadership in Applied Research and Care (CLARHC) in Leicestershire, Northamptonshire and Rutland. The CLARHC is funded by the NIHR. The views expressed in this paper do not necessarily reflect those of the NIHR. The funders had no role in study design, data collection and analysis, decision to publish, or preparation of the manuscript.

Contributor Statement

The study was conceived by KH, RB, JB and DJ. KH designed the study, carried out the analysis and drafted the initial manuscript. JB and DJ contributed to the statistical analysis. RB, JB and DJ contributed to drafting and editing the final manuscript and interpreting and reviewing the results of the statistical analysis.

Copyright Statement

The Corresponding Author has the right to grant on behalf of all authors and does grant on behalf of all authors, a worldwide licence (http://www.bmj.com/sites/default/files/BMJ%20Author%20Licence%20March%202013.doc) to the Publishers and its licensees in perpetuity, in all forms, formats and media (whether known now or created in the future), to i) publish, reproduce, distribute, display and store the Contribution, ii) translate the Contribution into other languages, create adaptations, reprints, include within collections and create summaries, extracts and/or, abstracts of the Contribution and convert or allow conversion into any format including without limitation audio, iii) create any other derivative work(s) based in whole or part on the on the Contribution, iv) to exploit all subsidiary rights to exploit all subsidiary rights that currently exist or as may exist in the future in the Contribution, v) the inclusion of electronic links from the Contribution to third party material where-ever it may be located; and, vi) licence any third party to do any or all of the above. All research articles will be made available on an Open Access basis (with authors being asked to pay an open access fee—see http://www.bmj.com/about-bmj/resources-authors/forms-policies-and-checklists/copyright-open-access-and-permission-reuse). The terms of such Open Access shall be governed by a Creative Commons licence—details as to which Creative Commons licence will apply to the research article are set out in our worldwide licence referred to above.

Ethical committee approval

Not required, as the study used only data that were already collected or publicly available, with no individuals or practices identified.

For peer review only

For peer review only - http://bmjopen.bmj.com/site/about/guidelines.xhtml

For peer review only

For peer review only - http://bmjopen.bmj.com/site/about/guidelines.xhtml

1 Department of Health. *Equity and excellence: Liberating the NHS*. London: Stationery Office, 2010. (White paper)

2 Department of Health. *The NHS Outcomes Framework 2013/14.* 2012. https://www.gov.uk/government/publications/nhs-outcomes-framework-2013-to-2014 (accessed 10 May 2013)

3 Townsend N, Wickramasinghe K, Bhatnagar P, *et al*. *Coronary heart disease statistics 2012 edition*. 2012. British Heart Foundation: London.

4 Murray CJ, Richards MA, Newton JN, *et al.* UK health performance: findings of the Global Burden of Disease Study 2010. *Lancet* 2013;**6736**. doi:10.1016/S0140-6736(13)60355-4

5 Macinko J, Starfield B, Shi L. Quantifying the health benefits of primary care physician supply in the United States. *Int J Health Serv* 2007;**37**:111–26.

6 Baker R, Sullivan E, Camosso-Stefinovic J, *et al.* Making use of mortality data to improve quality and safety in general practice: a review of current approaches. *Qual Saf Health Care* 2007;**16**:84–9.

7 Ashworth M, Schofield P, Doran T, *et al.* The Public Health Impact score: a new measure of public health effectiveness for general practices in England. *Br J Gen Pract* 2013;**63**:291–9.

8 Levene LS, Bankart J, Khunti K, *et al.* Association of primary care characteristics with variations in mortality rates in England: an observational study. *PLoS ONE* 2012;**7**(10):e47800. doi:10.1371/journal.pone.0047800

9 Baker R, Jones DR, Goldblatt P. Monitoring mortality rates in general practice after Shipman. *BMJ* 2003;**326**:274–6.

10 Kiran T, Hutchings A, Dhalla IA, *et al.* The association between quality of primary care, deprivation and cardiovascular outcomes: a cross-sectional study using data from the UK Quality and Outcomes Framework. *Epidemiol Community Health* 2010;**64**:927–34.

11 Levene LS, Baker R, Bankart MJG, *et al.* Association of features of primary health care with coronary heart disease mortality. *JAMA* 2010;**304**:2028–34.

12 Bottle A, Gnani S, Saxena S, *et al.* Association between quality of primary care and hospitalization for coronary heart disease in England: national cross-sectional study. *J Gen Intern Med* 2008;**23**:135–41.

13 Chauhan M, Bankart MJ, Labeit A, *et al.* Characteristics of general practices associated with numbers of elective admissions. *J Public Health* 2012;**34**:584–90.

For peer review only - http://bmjopen.bmj.com/site/about/guidelines.xhtml

For peer review only

14 Bankart MJG, Baker R, Rashid a, *et al.* Characteristics of general practices associated with emergency admission rates to hospital: a cross-sectional study. *Emerg Med J* 2011;**28**:558–63.

15 Rosenbaum PR, Rubin DB. Rates of Age-Adjusted Analyses Regression with Difficulties. *Biometrics* 1984;**40**:437–43.

16 Guo H-R. Age adjustment in ecological studies: using a study on arsenic ingestion and bladder cancer as an example. *BMC Public Health* 2011;**11**:820. doi:10.1186/1471-2458-11-820

17 Milyo J, Mellor JM. Income Inequality and Health Status in the United States Evidence from the Current Population Survey. *J Hum Resour* 2002;**37**:510–39.

18 Gardner W, Mulvey EP, Shaw EC. Regression analyses of counts and rates: Poisson, overdispersed Poisson, and negative binomial models. *Psychol Bull* 1995;**118**:392–404.

19 Freemantle N, Wood J, Griffin C, *et al.* What factors predict differences in infant and perinatal mortality in primary care trusts in England? A prognostic model. *BMJ* 2009;**339**:b2892–b2892.

20 Alter DA, Stukel TA, Newman A. The relationship between physician supply, cardiovascular health service use and cardiac disease burden in Ontario: Supply-need mismatch. *Can J Cardiol* 2008;**24**:187–93.

21 Bankart MJG, Baker R, Rashid a, *et al.* Characteristics of general practices associated with emergency admission rates to hospital: a cross-sectional study. *Emerg Med J* 2011;**28**:558–63.

22 Wheller L, Baker A, Griffiths C. Trends in premature mortality in England. *Health Stat Q* 2006;**31**:34-41

23 Allender S, Scarborough P, Keegan T, *et al.* Relative deprivation between neighbouring wards is predictive of coronary heart disease mortality after adjustment for absolute deprivation of wards. *J Epidemiol Community Health* 2012;**66**:803–8.

24 Lakhani A, Olearnik H, Eayres D. *Compendium of Clinicial and Helath Indicators: Data Definitions and User Guide for Computer File* 2011. The NHS Information Centre for health and social care. https://groups.ic.nhs.uk/archivedcompendia/public/Documents/June 2011/2011_06_User_Guide.pdf. (accessed 10 May 2013)

25 Quality and Outcomes Framework. *The Health and Social Care Information Centre: QOF online results database*. 2011 [online] Available at: http://www.qof.ic.nhs.uk [Accessed: 11 May 2013].

26 Dapartment of Health. *The GP Patient Survey*. 2007 [online] Available at: http://www.gp-patient.co.uk/download/Questionnaires/Y1 GP Patient Survey Questionnaire.pdf

27 Coleman T. Do financial incentives for delivering health promotion counselling work? Analysis of smoking cessation activities stimulated by the quality and outcomes. *BMC public health* 2010;**10**(167) doi:10.1186/1471-2458-10-1

For peer review only - http://bmjopen.bmj.com/site/about/guidelines.xhtml

28 Soljak M, Calderon-Larrañaga A, Sharma P, *et al.* Does higher quality primary health care reduce stroke admissions? *Br J Gen Pract* 2011;**61**(593):e801-7. doi:10.3399/bjgp11X613142.

29 Wolinsky FD, Bentler SE, Liu L, *et al.* Continuity of care with a primary care physician and mortality in older adults. *J Gerontol A Biol Sci Med Sci* 2010;**65**:421–8.

30 Downing A, Rudge G, Cheng Y, *et al.* Do the UK government's new Quality and Outcomes Framework (QOF) scores adequately measure primary care performance? A cross-sectional survey of routine healthcare data. *BMC Health Serv Res* 2007;**7**:166-172.

31 Soljak M, Samarasundera E, Indulkar T, *et al.* Variations in cardiovascular disease under-diagnosis in England: national cross-sectional spatial analysis. *BMC Cardiovasc Disord* 2011;**11**:12.

For peer review only

For peer review only - http://bmjopen.bmj.com/site/about/guidelines.xhtml

APPENDIX 1: Effect of choice of measure.

Table A1:1 Estimated incident rate ratios, associated 95% confidence intervals and associated *p* values for negative binomial regression for premature (U75) CHD mortality count.

	IRR	95% CI	*p* value
**Percentage white patients**	1.007	(1.003, 1.012)	0.002
**Deprivation score**	1.018	(1.012, 1.024)	<0.001
**Prevalence of diabetes 2006/07**	1.114	(1.029, 1.207)	0.008
**Percentage over 65**	1.051	(1.030, 1.072)	<0.001
**Number of GPs per 1000 patients**	1.173	(0.866, 1.589)	0.304
**Percentage male patients**	1.063	(1.028, 1.098)	<0.001
**Prevalence of hypertension 2006/07**	0.981	(0.953, 1.009)	0.182
**CHD quality achievement score (after Kiran et al)**	0.988	(0.978, 0.999)	0.037
**% of patients with recalled perception of being able to see preferred GP**	0.995	(0.989, 1.000)	0.061

APPENDIX 2: Effect of model choice

Table A2:1 Estimated regression coefficients for **weighted linear regression** showing the association between two CHD indicators and indirectly standardised **premature** (U75) CHD mortality rate.

	Coefficients	95% CI	*p* value
**Percentage white patients**	0.36	-0.08 to 0.80	0.105
**Deprivation score**	2.30	1.59 to 3.01	<0.001
**Prevalence of diabetes 2006/07**	2.53	-5.69 to 10.75	0.545
**List size**	-0.00038	-0.00025 to 0.00032	0.790
**Percentage over 65**	2.06	0.13 to 3.99	0.037
**Number of GPs per 1000 patients**	16.63	-15.39 to 48.64	0.307
**Percentage male patients**	2.95	-0.78 to 6.68	0.121
**Prevalence of hypertension 2006/07**	-2.74	-5.61 to 0.14	0.062
**% patients offered smoking cessation advice (SM02)**	0.67	-0.15 to 1.50	0.111
**% serum cholesterol (CHD08)**	-0.24	-1.15 to 0.67	0.607
**% aspirin (CHD09)**	0.24	-1.47 to 1.95	0.782
**% of patients with recalled perception of being able to see preferred GP**	-0.88	-1.43 to -0.33	0.002
**R^2**	0.31		

Table A2: 2 Estimated regression coefficients for weighted linear regression showing the association between 'Kiran' CHD quality achievement score and indirectly standardised **premature** (U75) CHD mortality rate.

	Coefficients	95% CI	*p* value
**Percentage white patients**	0.34	-0.110 to 0.78	0.132
**Deprivation score**	2.32	1.62 to 3.03	<0.001
**Prevalence of diabetes 2006/07**	2.30	-5.94 to 10.53	0.583
**List size**	-0.000057	-0.00022 to 0.00034	0.688
**Percentage over 65**	1.79	-0.11 to 3.69	0.064
**Number of GPs per 1000 patients**	16.63	-15.47 to 48.72	0.308
**Percentage male patients**	2.72	-1.00 to 6.45	0.151
**Prevalence of hypertension 2006/07**	-2.51	-5.37 to 0.34	0.084
**CHD quality achievement score (after Kiran et al)**	-0.20	-0.85 to 1.25	0.711
**% of patients with recalled perception of being able to see preferred GP**	-0.865	-1.41 to -0.32	0.002
**R^2**	0.31		

For peer review only

APPENDIX 3: Comparison with all age mortality

Table A3:1 Estimated incident rate ratios and associated 95% confidence intervals and significance levels for negative binomial regression for two CHD indicators and all age CHD mortality rate.

	IRR	*95% CI*	*P value*
**Percentage white patients**	1.007	(1.003, 1.010)	<0.001
**Deprivation score**	1.013	(1.008, 1.017)	<0.001
**Prevalence of diabetes 2006/07**	1.089	(1.028, 1.153)	0.004
**Percentage over 65**	1.074	(1.059, 1.089)	<0.001
**Number of GPs per 1000 patients**	1.039	(0.830, 1.279)	0.725
**Percentage male patients**	1.008	(0.984, 1.033)	0.598
**Prevalence of hypertension 2006/07**	0.989	(0.969, 1.008)	0.249
**% patients offered smoking cessation advice (SM02)**	1.005	(0.999, 1.011)	0.117
**% serum cholesterol (CHD08)**	0.993	(0.986, 1.000)	0.036
**% aspirin (CHD09)**	0.995	(0.981, 1.009)	0.454
**% of patients with recalled perception of being able to see preferred GP**	0.998	(0.994, 1.002)	0.418

Table A3: 2 Estimated incident rate ratios and associated 95% confidence intervals and significance levels for negative binomial regression for 'Kiran' CHD quality achievement score and all age CHD mortality rate.

	**IRR**	**95% CI**	***p* Value**
**Percentage white patients**	1.006	(1.003, 1.010)	<0.001
**Deprivation score**	1.013	(1.009, 1.018)	<0.001
**Prevalence of diabetes 2006/07**	1.086	(1.024, 1.150)	0.005
**Percentage over 65**	1.074	(1.059, 1.089)	<0.001
**Number of GPs per 1000 patients**	1.022	(0.822, 1.271)	0.844
**Percentage male patients**	1.006	(0.982, 1.031)	0.639
**Prevalence of hypertension 2006/07**	0.990	(0.971, 1.010)	0.334
**CHD quality achievement score (after Kiran et al)**	0.992	(0.984, 0.999)	0.033
**% of patients with recalled perception of being able to see preferred GP**	0.999	(0.995, 1.003)	0.564

Table A3:3 Estimated regression coefficients for weighted linear regression showing the association between two CHD indicators and indirectly standardised all age CHD mortality rate.

	**Coefficients**	**95% CI**	***p* Value**
**Percentage white patients**	0.45	0.14 to 0.75	0.004
**Deprivation score**	1.33	0.87 to 1.80	<0.001
**Prevalence of diabetes 2006/07**	6.08	0.65 to 11.52	0.028
**List size**	0.000052	-0.00015 to 0.00025	0.610
**Percentage over 65**	0.269	-1.07 to 1.61	0.693
**Number of GPs per 1000 patients**	5.54	-16.65 to 27.72	0.623
**Percentage male patients**	-2.37	-4.63 to -0.114	0.040
**Prevalence of hypertension 2006/07**	-1.24	-3.25 to 0.77	0.225
**% patients offered smoking cessation advice (SM02)**	0.38	-0.21 to 0.97	0.205
**% serum cholesterol (CHD08)**	-0.34	-0.95 to 0.27	0.275
**% aspirin (CHD09)**	-0.80	-2.10 to 0.50	0.227
**% of patients with recalled perception of being able to see preferred GP**	-0.20	-0.57 to 0.17	0.284
**R^2**	0.32		

Table A3:4 Estimated regression coefficients for weighted linear regression showing the association between 'Kiran' CHD quality achievement score and indirectly standardised all age CHD mortality rate.

	**Coefficients**	**95% CI**	***p* Value**
**Percentage white patients**	0.44	0.13 to 0.75	0.005
**Deprivation score**	1.37	0.91 to 1.83	<0.001
**Prevalence of diabetes 2006/07**	6.25	0.79 to 11.71	0.025
**List size**	0.000034	-0.00017 to 0.00023	0.736
**Percentage over 65**	0.317	-1.01 to 1.64	0.638
**Number of GPs per 1000 patients**	5.44	-16.92 to 27.79	0.632
**Percentage male patients**	-2.71	-4.95 to -0.46	0.018
**Prevalence of hypertension 2006/07**	-0.916	-2.835 to 1.003	0.348
**CHD quality achievement score (after Kiran et al)**	-0.382	-1.12 to 0.360	0.311
**% of patients with recalled perception of being able to see preferred GP**	-0.178	-0.55 to 0.195	0.348
**R^2**	0.31		

For peer review only

STROBE 2007 (v4) Statement—Checklist of items that should be included in reports of *cross-sectional studies*

Section/Topic	Item #	Recommendation	Reported on page #
**Title and abstract**	1	(*a*) Indicate the study's design with a commonly used term in the title or the abstract	Title page
		(*b*) Provide in the abstract an informative and balanced summary of what was done and what was found	2
**Introduction**			
Background/rationale	2	Explain the scientific background and rationale for the investigation being reported	4 & 5
Objectives	3	State specific objectives, including any prespecified hypotheses	2 - abstract
**Methods**			
Study design	4	Present key elements of study design early in the paper	6
Setting	5	Describe the setting, locations, and relevant dates, including periods of recruitment, exposure, follow-up, and data collection	6
Participants	6	(*a*) Give the eligibility criteria, and the sources and methods of selection of participants	6
Variables	7	Clearly define all outcomes, exposures, predictors, potential confounders, and effect modifiers. Give diagnostic criteria, if applicable	6
Data sources/ measurement	8*	For each variable of interest, give sources of data and details of methods of assessment (measurement). Describe comparability of assessment methods if there is more than one group	6 & 7 & Table 2 p13
Bias	9	Describe any efforts to address potential sources of bias	*6 & 7*
Study size	10	Explain how the study size was arrived at	6
Quantitative variables	11	Explain how quantitative variables were handled in the analyses. If applicable, describe which groupings were chosen and why	6 & 7
Statistical methods	12	(*a*) Describe all statistical methods, including those used to control for confounding	7
		(*b*) Describe any methods used to examine subgroups and interactions	n/a
		(*c*) Explain how missing data were addressed	7
		(*d*) If applicable, describe analytical methods taking account of sampling strategy	n/a
		(*e*) Describe any sensitivity analyses	7 & 8
**Results**			

For peer review only

Participants	13*	(a) Report numbers of individuals at each stage of study—eg numbers potentially eligible, examined for eligibility, confirmed eligible, included in the study, completing follow-up, and analysed	6
		(b) Give reasons for non-participation at each stage	n/a
		(c) Consider use of a flow diagram	n/a
Descriptive data	14*	(a) Give characteristics of study participants (eg demographic, clinical, social) and information on exposures and potential confounders	Table 1 p13<br>Table 3 p14
		(b) Indicate number of participants with missing data for each variable of interest	n/a
Outcome data	15*	Report numbers of outcome events or summary measures	Table 3 p14
Main results	16	(*a*) Give unadjusted estimates and, if applicable, confounder-adjusted estimates and their precision (eg, 95% confidence interval). Make clear which confounders were adjusted for and why they were included	Table 1 p13<br>Table 3 p14
		(*b*) Report category boundaries when continuous variables were categorized	n/a
		(*c*) If relevant, consider translating estimates of relative risk into absolute risk for a meaningful time period	n/a
Other analyses	17	Report other analyses done—eg analyses of subgroups and interactions, and sensitivity analyses	7 & 8 & 9
**Discussion**			
Key results	18	Summarise key results with reference to study objectives	8 & 9
Limitations	19	Discuss limitations of the study, taking into account sources of potential bias or imprecision. Discuss both direction and magnitude of any potential bias	10
Interpretation	20	Give a cautious overall interpretation of results considering objectives, limitations, multiplicity of analyses, results from similar studies, and other relevant evidence	9 & 11
Generalisability	21	Discuss the generalisability (external validity) of the study results	11
**Other information**			
Funding	22	Give the source of funding and the role of the funders for the present study and, if applicable, for the original study on which the present article is based	16

*Give information separately for cases and controls in case-control studies and, if applicable, for exposed and unexposed groups in cohort and cross-sectional studies.

Note: An Explanation and Elaboration article discusses each checklist item and gives methodological background and published examples of transparent reporting. The STROBE checklist is best used in conjunction with this article (freely available on the Web sites of PLoS Medicine at http://www.plosmedicine.org/, Annals of Internal Medicine at http://www.annals.org/, and Epidemiology at http://www.epidem.com/). Information on the STROBE Initiative is available at www.strobe-statement.org.

For peer review only

TITLE PAGE

Title: ~~Modelling factors in primary care affecting premature coronary heart disease mortality: a cross sectional study.~~

Modelling factors in primary care quality improvement: a cross sectional study of premature CHD mortality.

Corresponding author:
Kate Honeyford MSc
Adrian Building,
University Road,
Leicester
LE1 7RH
ceh28@le.ac.uk
Tel: 0116 229 7254/7255
Fax: 0116 229 7250

Co-authors:
Professor Richard Baker, -Dept of Health Sciences, University of Leicester, Leicester, UK.
Dr M. John G. Bankart, Insitute of Primary Care and Health Sciences, Keele University, Keele, UK.
Professor David Jones, Dept Health Sciences, University of Leicester, Leicester, UK.

Key words:
Primary health care
Regression analysis
Mortality, premature
Coronary disease
Cross-sectional studies

Word count: 3995 (including section headings)

For peer review only

For peer review only - http://bmjopen.bmj.com/site/about/guidelines.xhtml

ABSTRACT

Objectives

i) To identify features of primary care quality improvement associated with improved health outcomes using premature coronary heart disease (CHD) mortality as an example, andTo identify features of primary care associated with reduced premature coronary heart disease (CHD) mortality, and

ii) to determine the impacts of different modelling approaches.

Design

Cross sectional study of mortality rates in 229 general practices.

Setting

General practices from three East Midlands primary care trusts.

Participants

Patients registered to practices above between April 2006 and March 2009.

Main outcome measures

Numbers of CHD deaths in those aged under 75 (premature mortality) and at all ages in each practice.

Results

Both population characteristics and markers of quality of primary care were associated with variations in premature CHD mortality. Increasing levels of deprivation, and the percentage of the percentages of practice populations on the practice diabetes registers, white, over 65 and male were all associated with increasing levels of premature CHD mortality. Control of serum cholesterol levels in those with CHD and the percentage of patients recalling access to their preferred GP were both associated with decreased levels of premature CHD mortality. Similar results were found for all-age mortality. A combined measure of quality of primary care with regards tofor CHD comprising 12 QOF indicators was associated with decreases in both all-age and premature CHD mortality. The selected models suggest that practices in less deprived areas may have up to 20% lower premature CHD mortality than those with median deprivation and that improvement in the CHD quality of care measurecare quality from 83% (lower quartile) to 86% (median) could reduce premature CHD mortality by 3.6%. Different modelling approaches yielded qualitatively similar results.

Conclusions

High quality primary care, including aspects of access to and continuity of care, detection and management, appears to be associated with reducing CHD mortality. The impact on premature CHD mortality is greater than on all-age CHD mortality. Determining the most useful measures of quality of primary care needs further consideration.

For peer review only - http://bmjopen.bmj.com/site/about/guidelines.xhtml

Article Focus:

- CHD is a major cause of premature deaths in the UK; primary care can play a role in modifying risk factors in populations.
- This study identifies features of primary care associated with lower levels of premature coronary heart disease.
- A variety of methods have been used to model associations; this paper explores the impact of different modelling approaches.

Key Messages:

- High quality primary care, as measured through QOF clinical indicators and relational continuity, are associated with lower levels of premature CHD mortality.
- To inform future monitoring and development of primary care, more reliable data relating to smoking and other lifestyle factors is necessary.
- Results and their interpretation show some sensitivity to modelling approaches.

Strengths and weaknesses

- The associations between several modifiable risk factors and premature CHD mortality are considered, and compared to those for all age CHD mortality.
- The study examines the impact of model choice and the measure of primary care quality on results and interpretation.
- The lack of reliable data relating to lifestyle factors such as smoking and obesity rates mean that important covariates are not included in the model.

For peer review only - http://bmjopen.bmj.com/site/about/guidelines.xhtml

INTRODUCTION

In England, in common with health systems in many other countries, health outcomes are increasingly being used to plan and manage health services.[1] A framework for health outcomes has been introduced to provide an overview of how the national health service (NHS) is performing and as a catalyst for driving quality.[2] One of the framework's five domains is the prevention of premature death, one of the twelve indicators in this domain being death from cardiovascular disease under the age of 75 years.

Whilst mortality caused by coronary heart disease (CHD) has been falling steadily since the 1980s[3] it is still the leading cause of years of life lost in the UK.[4] ~~A recent report based on the~~The Global Burden Disease Study[4] shows that CHD mortality rates in the UK are significantly above the mean of ~~the 15 original members of the European Union and four other~~ those in comparator countries. Although population characteristics including lifestyle (eg obesity, smoking) and hypertension are known to be associated with CHD mortality, primary care may have a role in reducing CHD mortality. For example, in the US, greater supply of primary care physicians is associated with lower heart disease mortality,[5] although there is little evidence of a similar association in the UK.[6] Since general practices serve lists of registered patients, there is potential to relate mortality in ~~these~~ practice populations to both population characteristics and ~~the~~ performance of practices, and thereby assist practices in planning and monitoring their activities to reduce population mortality.[7] A conceptual model ~~to describe~~describing how the delivery of primary care can modify the impact of population based characteristics on health outcomes has been proposed.[8] ~~The model~~ It includes interventions which target both the morbid and healthy populations via early detection, prevention and appropriate management of people with established disease and highlights the importance of access and continuity of care. However, the size of practice populations and the small numbers of deaths present methodological difficulties.[9] In seeking to understand how primary care performance affects mortality in practice populations, consideration must be given to **(1)** the ~~approach to~~ measurement of the performance of primary care; and **(2)** selection of the underlying statistical model.

In recent years, information about the performance of general practices has become available, in addition to other practice characteristics such as numbers of registered patients and numbers of general practitioners. The Quality Outcomes Framework (QOF) has provided data in four domains (clinical; organisational; patient experience and additional). Within the clinical domain are several chronic conditions (including ~~coronary heart disease~~CHD), each made up of individual indicators. For each indicator points are awarded to practices based on the percentage of patients for whom the target has been achieved (known as the underlying achievement). ~~Both the s~~Score and ~~the~~ underlying achievement for each indicator are publicly available. Additionally, QOF contains 'prevalence registers' ~~that show~~showing the percentage of the practice population identified by the practice as having a particular condition or disease.

In investigating aspects of practice performance associated with various outcomes, Kiran et al used combinations of individual indicators' underlying achievement to give a devised 'CHD quality achievement score'.[10]

For peer review only - http://bmjopen.bmj.com/site/about/guidelines.xhtml

QOF prevalence registers have also been used as a measures of quality of primary care; for example, in a study at the level of primary care trusts, greater numbers of people on practice hypertension registers in primary care trusts (PCTs) have been shown to be associated with lower CHD mortality, indicating that improved detection of hypertension has a positive impact on outcome.[8 11]

The different ways in which QOF data have been used may partly explain the differences found in different studies.Differences between study results may be partly explained by different usage of QOF data. For example, Kiran et al [10] found that the overall CHD quality achievement score was significantly negatively associated with CHD mortality. However, other studies have shown no association between *individual* indicators and CHD mortality [8] and CHD admissions [12] or overall scores and emergency and elective admissions. [13 14]

Statistical Method and Model Selection

Different statistical models may be used. A common method is multiple linear regression with age-standardised mortality rates as the dependent variable. However, since in research of this type age-adjusted variables are rarely available multiple linear regression of standardised mortality rates can lead to biased risk estimates.[15–17]

Poisson models have the advantage of considering deaths as count data; age and sex can then be included in the model as explanatory variables, thus overcoming the lack of age standardised explanatory variables. As the counts are often over-dispersed, a negative binomial model may be more appropriate than a Poisson model.[18] Models based on count data, for example the numbers of deaths or hospital admissions, can also be found in the literature.[8 13 19–21]

Premature Mortality

Premature mortality may be defined in various ways, although the Outcomes Framework adopts a definition of deaths under age 75, in accordance with the preference of the Office of National Statistics.This study defines premature mortality in those under 75, in line with the Outcomes Framework and Office of National Statistics preferences.[22] Few studies have considered the associations between quality of primary health care, as measured by QOF, and *premature* mortality. Allender et al[23] demonstrated a stronger association between socio-economic deprivation and premature CHD mortality, compared to all-age CHD mortality. In a study of CHD hospitalisation and primary care[12] patients were divided into 45-74 and 75 and over; socio-economic status was found to be more important in the younger age group, but no difference was found in the association of quality of care and health outcomes in the two different age groups.

In this study, we investigate the choice of performance indicators and statistical models to explain all-age and premature premature and all-age practice population mortality from coronary heart diseaseCHD. The overall aim is to explore approaches with potential to help practices plan strategies to

For peer review only

~~reduce population mortality.~~The overall aim is to identify key aspects of primary care quality improvement having potential to improve health outcomes, here to reduce premature CHD mortality.

For peer review only - http://bmjopen.bmj.com/site/about/guidelines.xhtml

METHODS

This is an observational study of CHD mortality between April 2006 and March 2009 in 229 practices in the East Midlands. All 230 practices that were open for the three years of the study were eligible for inclusion. One practice offering a service to a restricted patient group was excluded from the study. The total population covered by the study is just under 1.7 million people. In this study premature CHD mortality will be the main outcome measure; this will be compared with all-age CHD mortality. Counts of deaths will be modelled using negative binomial regression. Results will be compared to those from weighted linear regression of indirectly standardised mortality ratios to determine the impact of the model adopted on the overall findings and conclusions of the analysis.

CHD mortality counts for each practice were constructed from the Primary Care Mortality Database for the period, supplied by the relevant PCTs. Each record included date of birth, date of death, underlying cause of death and the general practice code for the patient's practice. Each death was linked to a general practice using these codes. Deaths with underlying cause identified as CHD (ICD-10 codes: I20-I25) were included in the study. In addition, indirectly standardised mortality rates (SMR) were calculated for each practice, based on England and Wales' standard rates and midyear estimates of practice populations provided by PCTs in line with NCHOD's methodology.[24] Since numbers of deaths in each practice per year were low, both counts and SMRs were aggregated over the three years.

QOF clinical data are publicly available for all practices in the sample for 2006/07, 2007/08 and 2008/09.[25] The 2006/7 indicators were used since the primary care described by indicators for that year will have had an impact on the largest proportion of patients included in the analysis. Levene et al[8] classify primary healthcare into prevention, early detection and appropriate management. QOF indicators can be classified following this approach:

Prevention: Prevention of smoking and obesity. Reducing the number of people who are smoking or are obese would be useful indicators of prevention; however neither of these is available for practice populations. The QOF indicator which details the percentage of patients having a range of conditions who have been offered smoking cessation advice is being used as a measure of prevention of smoking (SM02).

Early detection: Detection of hypertension. Levene et al [8 11] argue that given only a little more than half of the people found to have hypertension in population surveys in England are included on QOF hypertension registers these can be used as a useful measure of hypertension detection.

Appropriate disease management: Indicators identified by Levene et al have been selected for this analysis:- **(a)** the control of serum cholesterol in patients on practice CHD registers (CHD08) and **(b)** the percentage of the practice CHD register who are being treated with aspirin (or equivalent) (CHD09).

See Table 1 for more detailed descriptions of QOF indicators.

For peer review only - http://bmjopen.bmj.com/site/about/guidelines.xhtml

The CHD quality achievement score devised by Kiran et al[10] is included in a comparative analysis. The measure is the mean underlying achievement of 12 indicators (see ~~details in~~ Table 1).

Access and sustained relationships: one question from the General Practice Patient Survey[26] relating to patients' recall of being able to consult a particular GP has been selected as an indicator of the ability to access care and whether patients prefer to have access to a sustained relationship in line with the work of Levene et al.[8] In addition, the number of GPs per 1000 patients is being included as a measure of GP supply.

Characteristics of the **practice population** have been selected based on previous research, the use of count data and availability. The percentage of the practice population who are male and are aged over 65 are included because they are known to be associated with higher rates of CHD mortality. List size is included as a measure of exposure in the negative binomial model, to take into ~~account that practices vary in size~~ variability in practice size and therefore the number at risk varies from practice to practice. List size is included in multiple regression for consistency. Diabetes prevalence, ethnicity and deprivation are included as previous research has shown these to be important in explaining variation in CHD mortality ([8 11], [10] and [8 10] respectively). ~~Neither obesity nor smoking rates are included as the measures available do not accurately describe the rates within practice populations.~~ Neither obesity nor smoking rates are included as the MSOA measures available are unlikely to describe rates within practice populations accurately.- The sources of information used for each of these are summarised in Table 2. Since CHD prevalence is highly correlated with both age and hypertension detection it was not included in the model to avoid collinearrity problems.

Eight practices have missing data for the number of GPs (fte); three practices have missing data from the GP Patient survey. The main analysis was repeated using a range of values; choice of value was not important. Either the median value or values from following years have been used.

Statistical analysis

Counts of deaths in each practice are modelled here using negative binomial regression in preference to Poisson regression since the data are over-dispersed (over-dispersion parameter α=0.023, $p<0.05$). To explore how sensitive the results and interpretation are to the modelling approach used, ordinary least squares (OLS) linear regression with the standardised mortality ratio (SMR) as the dependent variable was also carried out, using the inverse of the variance of SMR as a weighting to account for uncertainty in the standardised mortality rates, in line with the work of Kiran et al.[10]

For peer review only - http://bmjopen.bmj.com/site/about/guidelines.xhtml

RESULTS

Table 3 shows summary data of counts of death, mortality rates, and characteristics of both practice populations and primary care for the 229 practices, serving approximately 1.7 million patients in 2006/07. Table 4 shows the estimates of incident rate ratios (IRRs) in the presented negative binomial model, including 95% confidence intervals and *p* values for all explanatory variables; the impact of a one unit increase in each explanatory variable is also included to aid the interpretation of IRRs. As in many models in this field reporting pseudo R^2, adjusted pseudo R^2 is low, although the predicted and observed counts are relatively close (Lin's concordance coefficient : 0.86). There is no pattern in the residuals and removal of identified outliers did not materially affect interpretation of the model.

Table 5 shows the potential impact that changes in population or service characteristics could have on premature CHD mortality, given the current model. For each explanatory variable the change from either the upper or lower quartile to the median is used to show how decreases in the 'risk' in the practice population or increases in the quality of primary care could impact on mortality, if the model is adequate and relationships causal.

Sensitivity analysis explored the impact of various smoking and obesity indicators. Inclusion of QOF registers or modelled estimates for local area obesity prevalence do not materially change the coefficients; there is no evidence that the model is improved by so doing. Similar results are found when both modelled estimates of smoking rates and QOF indicators relating to smoking are included. None of these indicators has been included in the final model.

Population Characteristics

The main predictors of variation in premature CHD mortality of the population characteristics included in the model were deprivation, age (percentage aged 65 and over), ~~the~~ percentage on the diabetes register and ~~the~~ percentage male. Table 5 suggests that practices in less deprived areas may have up to 20% lower premature CHD mortality counts than those with deprivation at the median.

Service Characteristics

Increases in levels of service in terms of the percentage of the CHD register whose serum cholesterol is controlled (CHD08), and the percentage of patients who recalled being able to see their preferred GP, were associated with lower levels of premature CHD mortality, as was (less clearly) increased hypertension detection (as measured through QOF hypertension prevalence registers).

Similarly, there is a suggestion that increases in GPs per 1000 patients and the percentage of the CHD register being treated with aspirin (or an alternative) are both associated with higher premature mortality. Table 5 allows interpretation of these key variables in the current model, and suggests that increasing the percentage of patients who recall being able to see their preferred GP from a lower performing practice (typically 80%) to a median performing practice (88%) may reduce premature CHD mortality counts by 4.8%. Similarly, increasing the percentage of patients on the CHD register for whom

For peer review only - http://bmjopen.bmj.com/site/about/guidelines.xhtml

their serum cholesterol is below 5mmol/l from a low performing practice (78%) to a median performing practice (82%) may reduce premature CHD mortality counts by 3.6%.

Effect of choice of measure of quality of primary care

Similar results are found when the Kiran CHD quality achievement score is used in the model instead of the two separate CHD quality indicators. In the subsequent model an improvement from 83% (lower quartile) mean achievement in the Kiran overall CHD achievement score to 86% (median) reduces CHD mortality counts by 3.6% (see Appendix 1 for more information).

Effect of model selection

When a weighted multiple linear regression model for standardised mortality ratio was used, the directions of associations were the same as those described above. However, the confidence intervals for the β-coefficients for both prevalence of diabetes and the CHD quality achievement measures now include zero, and hence interpretation of the results might be less clear cut (see Appendix 2 for more information).

Comparison with all age mortality

The results for all age CHD mortality are broadly similar to those for premature CHD mortality. Increases in white ethnicity, deprivation and diabetes prevalence and an increase in GPs per 1000 patients are associated with increases in mortality, whereas improvements in the remaining service characteristics correspond to decreases in mortality counts. However, the confidence intervals for the IRRs for all service characteristics include one, impacting on the interpretation of the importance of these variables.

DISCUSSION

Statement of principal findings

Both population characteristics and the quality of primary care were found to be associated with variations in premature CHD mortality. Increasing levels of deprivation, the percentage of the practice population who were on the practice diabetes register, who were white, over 65 and who were male were all associated with increasing levels of premature CHD mortality. Control of serum cholesterol levels in those with CHD and the percentage of patients who could recall being able to see their preferred GP were both associated with decreased levels of premature CHD mortality. Similar results were found when all age mortality was considered. The combined measure of the quality of primary care was associated with a decrease in both all-age and premature CHD mortality. However, it is difficult to determine which individual indicators within this measure are key to reducing CHD mortality. The evidence that hypertension detection is associated with decreased CHD mortality is less clear than has been found by Levene et al at PCT level.[8] Different modelling approaches yielded qualitatively similar

For peer review only - http://bmjopen.bmj.com/site/about/guidelines.xhtml

For peer review only

results; however, detailed interpretation of the results would be model dependent, particularly if statistical significance were rigidly applied as a criterion of importance.

Strengths and weaknesses of this study

A key strength of this study is that it considers the association of features of primary care with *premature* CHD mortality, and whilst the overall relationships are similar to those found when all age mortality is considered, some associations are stronger, for example, that with patients' recall of being able to see their preferred GP.

Most explanatory variables in this study describe the adult practice population and not those under 75. To develop our understanding of the different contributions made by different variables in explaining premature mortality it would be helpful to have reliable data describing different age groups within practice populations.

This study allows useful consideration of the impact of the measure of primary care quality used. Whilst the higher underlying achievement in overall CHD achievement score devised by Kiran is associated with decreased premature mortality, this doesn't allow policy makers or clinicians to determine which of the 12 indicators are most important. The examination of the individual indicators shows that increasing the percentage of patients on the CHD register whose serum cholesterol is below 5mmol/l is associated with decreasing mortality, but it is ~~not clear~~unclear whether this is the most important indicator. Unfortunately, as patient level data relating to QOF indicators are not available for this study, it is not possible to determine whether combinations of indicators within individuals are key or if there are interactions between indicators and other characteristics of individuals, for example ethnicity or gender. Reliable information about smoking rates, alcohol consumption and obesity within practice populations is lacking, and having access to ~~this~~ these data would be likely to improve the model fit. Whilst various estimates are available, many of these are modelled estimates for geographical areas, not practice populations, based on levels of deprivation, ethnicity and age, which are anyway included in the models in this study. Integrated Household Study smoking data is available at local area level, and could be used to estimate smoking prevalence in practice populations. However, it was not possible to match these to practice populations for this study. The introduction of new smoking indicators in the 2012/13 QOF asks practices to record the smoking status of those aged 15 years and over and to record an offer of support and treatment to those who smoke, which may start to give a fuller picture of smoking prevalence in practice populations. Whilst a QOF smoking indicator relating to smoking cessation advice has been included in this study as a measure of prevention, the wording asks only for 'an offer of support and treatment' which may throw doubt on its validity.[27] White ethnicity percentage was included in the model; it is highly correlated (R_p: -0.993) with the percentage who are Asian. More refined ethnicity information was not available for thie study.

This study allows the impact of statistical method and model selection to be considered. Whilst the directions of associations are generally not affected by the choice of model the statistical significance of

For peer review only - http://bmjopen.bmj.com/site/about/guidelines.xhtml

the results vary between models for several key variables. For example, neither the prevalence of diabetes nor the CHD quality achievement score would be considered to be significantly associated with premature mortality using a weighted linear regression model for SMRs.

Relation to other studies

Previous research has shown that primary care is important in improving health outcomes but that the precise aspects of primary care which are most important are not clear.[8 10 13 14 28] This study confirms that position, but explores other aspects of the problem.

An association between continuity of care and reduced mortality in older patients has been found in the US[29] and Bankart et al[14] have shown that in practices with higher mean rates of satisfaction with being able to consult a preferred GP, emergency admissions are lower. These findings give further support to the importance of continuity of care in improving health outcomes.
Levene et al have found that hypertension detection was associated with reduced levels of CHD mortality in two studies completed at PCT level. In their ~~recent analysis of UK health performance based on the~~ Global Burden of Disease Study 2010 Murray et al[4] highlight the importance of early detection and long term management of high blood pressure as a 'clear route to accelerate progress for the leading causes of avoidable cardiovascular mortality'. However, the evidence of the importance of hypertension detection in this study is not clear cut. It may be that QOF prevalence registers are not a useful measure of detection for individual practices. Low QOF prevalence rates may be due to lower levels of hypertension rather than lower detection and conversely high QOF prevalence rates may be due to higher underlying prevalence rather than improved detection. More work to identify the most useful combination of QOF indicators to summarise hypertension detection is necessary.

~~In line~~Consistent with other studies, socio-economic deprivation is the main predictor of CHD mortality. Both Bottle[12] and Allender[23] have found that socio-economic deprivation is more strongly associated with mortality in younger age groups and this study has confirmed this pattern.

Whilst some studies clearly state the reasons for their selection of statistical modelling approach and/or potential explanatory variables (for example [13 14 19 30 31]), many studies do not. Here we find some indication that qualitative interpretations of results are robust to model choice. However, more detailed interpretation of results is likely to vary between models, particularly if there is undue reliance on simplistic interpretation of statistical significance.

Meaning of the study

This study adds to the body of research demonstrating that high quality primary care is associated with improving health outcomes. Aspects of both continuity of care and disease management have been identified as having a bigger impact on reducing premature CHD mortality than all-age CHD mortality. Whilst the most important individual indicators relating to disease management have not been identified, there is clear evidence that improving achievement in QOF indicators is associated with

For peer review only - http://bmjopen.bmj.com/site/about/guidelines.xhtml

For peer review only

decreasing CHD mortality. The importance of continuity of care, again shown here, strongly suggests that this is an area for general practices to prioritise. The findings also suggest that data about outcomes such as premature CHD mortality could be used by practices to monitor and, over several years, plan their care to improve population health.

The ongoing importance of socio-economic deprivation in explaining higher levels of mortality cannot be ignored. Understanding the relationship between deprivation and health outcomes more precisely remains an important area of further study.

Unanswered questions and future research

The lack of reliable practice level information on key areas such as obesity, alcohol and smoking prevalence has an important impact on primary care research and is an important health information issue needing effective attention as the NHS undergoes major changes. Whilst QOF indicators relating to smoking prevalence have been introduced, the reliability of these measures will need to be scrutinised. To further our understanding of the relative importance of different QOF indicators it would be useful to study individual QOF indicators at patient level and how they interact with each other and with characteristics of individuals on an individual patient basis. In future studies, the impact of the introduction of health checks in 2009 should be explored.

CONCLUSION

Improving the quality of primary care will play an important part in decreasing premature mortality, and there is evidence that high underlying achievements in QOF clinical indicators are a useful measure of quality primary care. Continuity of care, in a country with universal access to healthcare, is important and should not be underestimated by policy makers and clinicians. Nonetheless lifestyle factors are also important, but our ability to study them adequately in primary care, or to evaluate the role of primary care in addressing them, is currently limited by the quality of measures ~~of them~~ at practice level. If primary care services, delivered by clinical commissioning groups are to be monitored and developed using the new NHS Outcomes Framework, better data and careful modelling and interpretation are vital.

For peer review only - http://bmjopen.bmj.com/site/about/guidelines.xhtml

Tables

Table 1: QOF indicators used to compile the overall CHD quality achievement score.

Clinical domain			Summary statistics in this study (median and interquartile range)
**CHD**	CHD06	The percentage of patients with CHD in whom the last blood pressure reading (measured in the previous 15 months) is ≤150/90 mm Hg.	90 (85, 93)
	CHD08	The percentage of patients with CHD whose last measured total cholesterol level (measured in the previous 15 months) is ≤5 mmol/l.	82 (78, 87)
	CHD09	The percentage of patients with CHD with a record in the previous 15 months that aspirin, an alternative antiplatelet therapy, or an anti-coagulant is being taken (unless a contraindication or adverse effects are recorded).	95 (93, 97)
	CHD10	The percentage of patients with CHD who are currently treated with a β-blocker (unless a contraindication or adverse effects are recorded).	73 (66, 81)
	CHD11	The percentage of patients with a history of myocardial infarction (diagnosed after 1 April 2003) who are currently treated with an angiotensin-converting enzyme inhibitor or angiotensin II antagonist.	92 (88, 97)
**Stroke and TIA**	STROKE06	The percentage of patients with TIA or stroke in whom the last blood pressure reading (measured in the previous 15 months) is ≤150/90 mm Hg.	88 (82, 92)
	STROKE08	The percentage of patients with TIA or stroke whose last measured total cholesterol level (measured in the previous 15 months) is ≤5 mmol/l.	76 (70, 83)
**Hypertension**	BP05	The percentage of patients with hypertension in whom the last blood pressure reading (measured in the previous 9 months) is ≤150/90 mm Hg.	77 (73, 83)
**Diabetes mellitus**	DM12	The percentage of patients with diabetes in whom the last blood pressure reading is ≤145/85 mm Hg.	80 (74, 85)
	DM17	The percentage of patients with diabetes whose last measured total cholesterol level (measured in the previous 15 months) is ≤5 mmol/l.	83 (77, 87)
	DM20	The percentage of patients with diabetes in whom the last HbA1C is ≤ 7.5 (or equivalent test/reference range depending on laboratory) in the previous 15 months.	67 (61, 73)
**Smoking**	SMOKE02	The percentage of patients with any or any combination of the following condition: CHD, stroke or TIA, hypertension, diabetes, COPD or asthma, who smoke and whose notes contain a record that smoking cessation advice or referral to a specialist service, where available, has been offered within the previous 15 months.	94 (92, 96)
**Kiran CHD overall achievement score**		Mean of the above 12 indicators	83 (80, 86)

Table 2: Characteristics of practice populations.

Measure	Source of data
**Deprivation indices**	Based on Index of Multiple Deprivation 2007.
**% of GP list on diabetes register**	QOF prevalence register 2006/07.
**% White ethnicity**	Based on hospital episode statistics.
**% of population who are over 65**	Based on mid-year practice population estimates provided by the PCT.
**% of population who are male**	Based on mid-year practice population estimates provided by the PCT.
**List size**	Based on mid-year practice population estimates provided by the PCT.

For peer review only - http://bmjopen.bmj.com/site/about/guidelines.xhtml

Table 3 Summary statistics for mortality counts, population characteristics and service characteristics.

	Summary statistics in this study Median (IQR)
**Mortality data**	
Indirectly Standardised CHD mortality	95.9 (78.3, 119.9)
Indirectly Standardised CHD mortality – under 75	92.4 (64.0, 135.6)
Crude rate per 1000	1.30 (0.95, 1.57)
Crude rate per 1000 – under 75	0.43 (0.28, 0.58)
**Practice characteristics**	
Deprivation indices	16.2 (10.0, 27.0)
% of GP list on diabetes register	3.8 (3.3, 4.4)
% White ethnicity	89.9 (77.5, 94.1)
% of population who are over 65	14.7 (12.1, 17.0)
% of population who are male	50.2 (49.5, 51.2)
List size	6435 (3749, 10319)
**Service characteristics**	
GPs per 1000	0.55 (0.47, 0.64)
% patients with recalled perception of being able to see preferred GP	88 (80, 93)
% of GP registered list on hypertension register	12.3 (11.0, 14.7)
The percentage of patients with any or any combination of the following conditions: coronary heart disease, stroke or TIA, hypertension, diabetes, COPD or asthma who smoke whose notes contain a record that smoking cessation advice or referral to a specialist service, where available, has been offered within the previous 15 months. SM02	94 (92, 96)
The percentage of patients with CHD whose last measured total cholesterol level (measured in the previous 15 months) is ≤5 mmol/l. CHD08	82 (78, 87)
The percentage of patients with CHD with a record in the previous 15 months that aspirin, an alternative antiplatelet therapy, or an anti-coagulant is being taken (unless a contraindication or adverse effects are recorded). CHD09	95 (93, 97)
CHD overall achievement score[10]	83 (80, 86)

For peer review only - http://bmjopen.bmj.com/site/about/guidelines.xhtml

Table 4 Estimated incident rate ratios, associated 95% confidence intervals and associated *p* values for negative binomial regression for premature (U75) CHD mortality count. Also included are the effects on premature CHD mortality count of a unit increase in the value of the explanatory variables.

Explanatory variable	*IRR*	*95% CI*	*p value*	*Effect of a 1% increase in explanatory variable on percentage change in premature mortality (CI).*	
**Percentage white patients**	1.008	(1.003, 1.012)	0.002	+0.8%	(0.3, 1.2)
**Deprivation score**	1.017	(1.011, 1.024)	<0.001	+1.7%	(1.1, 2.3)
**Prevalence of diabetes 2006/07**	1.114	(1.028, 1.208)	0.009	+11.4%	(2.8, 20.8)
**Percentage over 65**	1.059	(1.038, 1.081)	<0.001	+5.9%	(3.8, 8.1)
**Percentage male patients**	1.067	(1.038, 1.103)	<0.001	+6.7%	(3.8, 10.3)
**Number of GPs per 1000 patients**	1.197	(0.885, 1.619)	0.244	+19.7%	(-11.5, 61.9)
**Hypertension detection 2006/07**	0.978	(0.950, 1.007)	0.133	-2.2%	(-5.0, 0.7)
**% patients offered smoking cessation advice (SM02)**	1.002	(0.993, 1.011)	0.712	+0.2%	(-0.7, 1.1)
**% serum cholesterol (CHD08)**	0.991	(0.981, 1.000)	0.044	-0.9%	(-1.9, 0.0)
**% aspirin (CHD09)**	1.002	(0.982, 1.022)	0.884	+0.2%	(-1.8, 2.2)
**% of patients with recalled perception of being able to see preferred GP**	0.994	(0.989, 1.00)	0.036	-0.6%	(-1.1, 0.0)

*(one unit on scale for deprivation score).

Table 5 Impact on premature (U75) CHD mortality count of an improvement in primary care or decrease in population burden to the median from the upper or lower quartile as appropriate, given the current model.

Explanatory variable	*Description of change*	*Effect of improvement in primary care or decrease in population burden on percentage change in premature mortality (CI).*
**Percentage white patients**	*Decrease from upper quartile to median – decrease of 5.2% in percentage white patients.*	*-4.16% (-6.24%, -1.56%)*
**Deprivation score**	*Decrease from upper quartile to median – decrease of 10.8 units on scale.*	*-18.36% (-25.92%, -11.88%)*
**Prevalence of diabetes 2006/07**	*Decrease from upper quartile to median – decrease of 0.6% in diabetes prevalence.*	*-6.84% (-12.48%, -1.68%)*
**Percentage over 65**	*Decrease from upper quartile to median – decrease of 2.3% in percentage over 65.*	*-13.57% (-18.63%, -8.74%)*
**Percentage male patients**	*Decrease from upper quartile to median – decrease of 1.0% in percentage male patients.*	*-6.7% (-10.3%, -3.8%)*
**Number of GPs per 1000 patients**	*Increase from lower quartile to median – increase of 0.8 GPs per 1000 patients.*	*15.76% (-9.20%, 49.52%)*
**Hypertension detection 2006/07**	*Increase from lower quartile to median – increase of 2.3% in detection.*	*-5.06% (-11.27%, 1.61%)*
**% patients offered smoking cessation advice (SM02)**	*Increase from lower quartile to median – increase of 2.06% offered advice*	*0.41% (-1.44%, 2.27%)*
**% serum cholesterol (CHD08)**	*Increase from lower quartile to median – increase of 4.0% in achieving serum cholesterol target.*	*-3.6% (-7.6%, 0.0%)*
**% aspirin (CHD09)**	*Increase from lower quartile to median – increase of 2.0% in aspirin treatment.*	*0.4% (-3.6%, 4.4%)*
**% of patients with recalled perception of being able to see preferred GP**	*Increase from lower quartile to median – increase of 8.0% in patients recalling being able to see preferred GP.*	*-4.8% (-8.8%, -0.00%)*

For peer review only - http://bmjopen.bmj.com/site/about/guidelines.xhtml

Acknowledgements, Competing Interests and Funding

Competing Interests

All authors have completed the ICMJE uniform disclosure form at www.icmje.org/coi_disclosure.pdf and declare: KH had financial support from CLAHRC in the form of funding for PhD fees; no financial relationships with any organisations that might have an interest in the submitted work in the previous three years; no other relationships or activities that could appear to have influenced the submitted work.

Funding

The study formed part of the programme of the National Institute for Health Research (NIHR) Collaboration for Leadership in Applied Research and Care (CLARHC) in Leicestershire, Northamptonshire and Rutland. The CLARHC is funded by the NIHR. The views expressed in this paper do not necessarily reflect those of the NIHR. The funders had no role in study design, data collection and analysis, decision to publish, or preparation of the manuscript.

Contributor Statement

The study was conceived by KH, RB, JB and DJ. KH designed the study, carried out the analysis and drafted the initial manuscript. JB and DJ contributed to the statistical analysis. RB, JB and DJ contributed to drafting and editing the final manuscript and interpreting and reviewing the results of the statistical analysis.

Copyright Statement

The Corresponding Author has the right to grant on behalf of all authors and does grant on behalf of all authors, a worldwide licence (http://www.bmj.com/sites/default/files/BMJ%20Author%20Licence%20March%202013.doc) to the Publishers and its licensees in perpetuity, in all forms, formats and media (whether known now or created in the future), to i) publish, reproduce, distribute, display and store the Contribution, ii) translate the Contribution into other languages, create adaptations, reprints, include within collections and create summaries, extracts and/or, abstracts of the Contribution and convert or allow conversion into any format including without limitation audio, iii) create any other derivative work(s) based in whole or part on the on the Contribution, iv) to exploit all subsidiary rights to exploit all subsidiary rights that currently exist or as may exist in the future in the Contribution, v) the inclusion of electronic links from the Contribution to third party material where-ever it may be located; and, vi) licence any third party to do any or all of the above. All research articles will be made available on an Open Access basis (with authors being asked to pay an open access fee—see http://www.bmj.com/about-bmj/resources-authors/forms-policies-and-checklists/copyright-open-access-and-permission-reuse). The terms of such Open Access shall be governed by a Creative Commons licence—details as to which Creative Commons licence will apply to the research article are set out in our worldwide licence referred to above.

Ethical committee approval

Not required, as the study used only data that were already collected or publicly available, with no individuals or practices identified.

For peer review only - http://bmjopen.bmj.com/site/about/guidelines.xhtml

For peer review only

For peer review only - http://bmjopen.bmj.com/site/about/guidelines.xhtml

1 Department of Health. *Equity and excellence: Liberating the NHS*. London: Stationery Office, 2010. (White paper)

2 Department of Health. *The NHS Outcomes Framework 2013/14.* 2012. https://www.gov.uk/government/publications/nhs-outcomes-framework-2013-to-2014 (accessed 10 May 2013)

3 Townsend N, Wickramasinghe K, Bhatnagar P, *et al*. *Coronary heart disease statistics 2012 edition*. 2012. British Heart Foundation: London.

4 Murray CJ, Richards MA, Newton JN, *et al.* UK health performance: findings of the Global Burden of Disease Study 2010. *Lancet* 2013;**6736**. doi:10.1016/S0140-6736(13)60355-4

5 Macinko J, Starfield B, Shi L. Quantifying the health benefits of primary care physician supply in the United States. *Int J Health Serv* 2007;**37**:111–26.

6 Baker R, Sullivan E, Camosso-Stefinovic J, *et al.* Making use of mortality data to improve quality and safety in general practice: a review of current approaches. *Qual Saf Health Care* 2007;**16**:84–9.

7 Ashworth M, Schofield P, Doran T, *et al.* The Public Health Impact score: a new measure of public health effectiveness for general practices in England. *Br J Gen Pract* 2013;**63**:291–9.

8 Levene LS, Bankart J, Khunti K, *et al.* Association of primary care characteristics with variations in mortality rates in England: an observational study. *PLoS ONE* 2012;**7**(10):e47800. doi:10.1371/journal.pone.0047800

9 Baker R, Jones DR, Goldblatt P. Monitoring mortality rates in general practice after Shipman. *BMJ* 2003;**326**:274–6.

10 Kiran T, Hutchings A, Dhalla IA, *et al.* The association between quality of primary care, deprivation and cardiovascular outcomes: a cross-sectional study using data from the UK Quality and Outcomes Framework. *Epidemiol Community Health* 2010;**64**:927–34.

11 Levene LS, Baker R, Bankart MJG, *et al.* Association of features of primary health care with coronary heart disease mortality. *JAMA* 2010;**304**:2028–34.

12 Bottle A, Gnani S, Saxena S, *et al.* Association between quality of primary care and hospitalization for coronary heart disease in England: national cross-sectional study. *J Gen Intern Med* 2008;**23**:135–41.

13 Chauhan M, Bankart MJ, Labeit A, *et al.* Characteristics of general practices associated with numbers of elective admissions. *J Public Health* 2012;**34**:584–90.

For peer review only - http://bmjopen.bmj.com/site/about/guidelines.xhtml

14 Bankart MJG, Baker R, Rashid a, *et al.* Characteristics of general practices associated with emergency admission rates to hospital: a cross-sectional study. *Emerg Med J* 2011;**28**:558–63.

15 Rosenbaum PR, Rubin DB. Rates of Age-Adjusted Analyses Regression with Difficulties. *Biometrics* 1984;**40**:437–43.

16 Guo H-R. Age adjustment in ecological studies: using a study on arsenic ingestion and bladder cancer as an example. *BMC Public Health* 2011;**11**:820. doi:10.1186/1471-2458-11-820

17 Milyo J, Mellor JM. Income Inequality and Health Status in the United States Evidence from the Current Population Survey. *J Hum Resour* 2002;**37**:510–39.

18 Gardner W, Mulvey EP, Shaw EC. Regression analyses of counts and rates: Poisson, overdispersed Poisson, and negative binomial models. *Psychol Bull* 1995;**118**:392–404.

19 Freemantle N, Wood J, Griffin C, *et al.* What factors predict differences in infant and perinatal mortality in primary care trusts in England? A prognostic model. *BMJ* 2009;**339**:b2892–b2892.

20 Alter DA, Stukel TA, Newman A. The relationship between physician supply, cardiovascular health service use and cardiac disease burden in Ontario: Supply-need mismatch. *Can J Cardiol* 2008;**24**:187–93.

21 Bankart MJG, Baker R, Rashid a, *et al.* Characteristics of general practices associated with emergency admission rates to hospital: a cross-sectional study. *Emerg Med J* 2011;**28**:558–63.

22 Wheller L, Baker A, Griffiths C. Trends in premature mortality in England. *Health Stat Q* 2006;**31**:34-41

23 Allender S, Scarborough P, Keegan T, *et al.* Relative deprivation between neighbouring wards is predictive of coronary heart disease mortality after adjustment for absolute deprivation of wards. *J Epidemiol Community Health* 2012;**66**:803–8.

24 Lakhani A, Olearnik H, Eayres D. *Compendium of Clinicial and Helath Indicators: Data Definitions and User Guide for Computer File* 2011. The NHS Information Centre for health and social care. https://groups.ic.nhs.uk/archivedcompendia/public/Documents/June 2011/2011_06_User_Guide.pdf. (accessed 10 May 2013)

25 Quality and Outcomes Framework. *The Health and Social Care Information Centre: QOF online results database*. 2011 [online] Available at: http://www.qof.ic.nhs.uk [Accessed: 11 May 2013].

26 Dapartment of Health. *The GP Patient Survey*. 2007 [online] Available at: http://www.gp-patient.co.uk/download/Questionnaires/Y1 GP Patient Survey Questionnaire.pdf

27 Coleman T. Do financial incentives for delivering health promotion counselling work? Analysis of smoking cessation activities stimulated by the quality and outcomes. *BMC public health* 2010;**10**(167) doi:10.1186/1471-2458-10-1

For peer review only - http://bmjopen.bmj.com/site/about/guidelines.xhtml

For peer review only

28 Soljak M, Calderon-Larrañaga A, Sharma P, *et al.* Does higher quality primary health care reduce stroke admissions? *Br J Gen Pract* 2011;**61**(593):e801-7. doi:10.3399/bjgp11X613142.

29 Wolinsky FD, Bentler SE, Liu L, *et al.* Continuity of care with a primary care physician and mortality in older adults. *J Gerontol A Biol Sci Med Sci* 2010;**65**:421–8.

30 Downing A, Rudge G, Cheng Y, *et al.* Do the UK government's new Quality and Outcomes Framework (QOF) scores adequately measure primary care performance? A cross-sectional survey of routine healthcare data. *BMC Health Serv Res* 2007;**7**:166-172.

31 Soljak M, Samarasundera E, Indulkar T, *et al.* Variations in cardiovascular disease under-diagnosis in England: national cross-sectional spatial analysis. *BMC Cardiovasc Disord* 2011;**11**:12.

For peer review only

For peer review only - http://bmjopen.bmj.com/site/about/guidelines.xhtml
